# SEAGLE: A Scalable Exact Algorithm for Large-Scale Set-Based Gene-Environment Interaction Tests in Biobank Data

**DOI:** 10.3389/fgene.2021.710055

**Published:** 2021-11-02

**Authors:** Jocelyn T. Chi, Ilse C. F. Ipsen, Tzu-Hung Hsiao, Ching-Heng Lin, Li-San Wang, Wan-Ping Lee, Tzu-Pin Lu, Jung-Ying Tzeng

**Affiliations:** ^1^ Department of Statistics, North Carolina State University, Raleigh, NC, United States; ^2^ Department of Mathematics, North Carolina State University, Raleigh, NC, United States; ^3^ Department of Medical Research, Taichung Veterans General Hospital, Taichung, Taiwan; ^4^ Penn Neurodegeneration Genomics Center, Department of Pathology and Laboratory Medicine, Perelman School of Medicine, University of Pennsylvania, Philadelphia, PA, United States; ^5^ Institute of Epidemiology and Preventive Medicine, National Taiwan University, Taipei, Taiwan; ^6^ Department of Statistics, National Cheng-Kung University, Tainan, Taiwan

**Keywords:** gene-based GxE test for biobank data, GxE collapsing test for biobank data, GxE test for large-scale sequencing data, scalable GEI test, gene-environment variance component test, gene-environment kernel test, regional-based gene-environment test

## Abstract

The explosion of biobank data offers unprecedented opportunities for gene-environment interaction (GxE) studies of complex diseases because of the large sample sizes and the rich collection in genetic and non-genetic information. However, the extremely large sample size also introduces new computational challenges in G×E assessment, especially for set-based G×E variance component (VC) tests, which are a widely used strategy to boost overall G×E signals and to evaluate the joint G×E effect of multiple variants from a biologically meaningful unit (e.g., gene). In this work, we focus on continuous traits and present SEAGLE, a **S**calable **E**xact **A**l**G**orithm for **L**arge-scale set-based G×**E** tests, to permit G×E VC tests for biobank-scale data. SEAGLE employs modern matrix computations to calculate the test statistic and *p*-value of the GxE VC test in a computationally efficient fashion, without imposing additional assumptions or relying on approximations. SEAGLE can easily accommodate sample sizes in the order of 10^5^, is implementable on standard laptops, and does not require specialized computing equipment. We demonstrate the performance of SEAGLE using extensive simulations. We illustrate its utility by conducting genome-wide gene-based G×E analysis on the Taiwan Biobank data to explore the interaction of gene and physical activity status on body mass index.

## 1 Introduction

Human complex diseases such as neurodegenerative diseases, psychiatric disorders, metabolic syndromes, and cancers are complex traits for which disease susceptibility, disease development, and treatment response are mediated by intricate genetic and environmental factors. Understanding the genetic etiology of these complex diseases requires collective consideration of potential genetic and environmental contributors. Studies of gene-environment interactions (G×E) enable understanding of the differences that environmental exposures may have on health outcomes in people with varying genotypes (Ottman, 1996; Hunter, 2005; McAllister et al., 2017). Examples include the impact of physical activity and alcohol consumption on the genetic risk for obesity-related traits (Sulc et al., 2020), the impact of air pollution on the genetic risk for cardio-metabolic and respiratory traits (Favé et al., 2018), and other examples reviewed in Ritz et al. (2017).

When assessing G×E effects, set-based tests are popular approaches to detecting interactions between an environmental factor and a set of single nucleotide polymorphism (SNPs) in a gene, sliding window, or functional region (Tzeng et al., 2011; Lin et al., 2013; Zhao et al., 2015; Lin et al., 2016; Su et al., 2017). Compared to single-SNP G×E tests, set-based G×E tests can enhance testing performance by reducing multiple-testing burden and by aggregating G×E signals over multiple SNPs that are of moderate effect sizes or of low frequencies.

Large-scale biobanks collect genetic and health information on hundreds of thousands of individuals. Their large sample sizes and rich data on non-genetic factors offer unprecedented opportunities for in-depth studies on G×E effects. While the explosion of biobank data collections provides great hopes for novel G×E discoveries, it also introduces computational challenges. In particular, many set-based G×E tests can be cast as variance component (VC) tests under a random effects modeling framework (Lin et al., 2013; Su et al., 2017), including kernel machine based tests (Wang et al., 2015a; Lin et al., 2016) and similarity regression based methods (Tzeng et al., 2011; Zhao et al., 2015). Hypothesis testing in this framework relies on computations with phenotypic variance matrices with dimension *n* × *n* (with *n* as the sample size) and may involve estimating nuisance variance components. When *n* is large, as in the case of biobank data, matrix computations whose operation counts scale with *n* ^3^ are prohibitive in terms of computation time and storage.

A number of methods attempt to ease this computational burden by bypassing the estimation of nuisance variance components, either through approximation of the variance or kernel matrices (Marceau et al., 2015) or through approximation of the score-like test statistics (Wang et al., 2020). In the first case, approximating the kernel matrices still requires an expensive eigenvalue decomposition upfront, in addition to storage for the explicit formation of the *n* × *n* kernel matrices, thus lacking practical scalability. In the latter case, approximating the test statistics requires assumptions that may or may not be valid and are difficult to validate in practice. Our numerical studies in Section 3 show that the Type 1 error rates and power can be sub-optimal when data do not adhere to the required assumptions.

In this work, we focus on continuous traits and introduce a Scalable Exact AlGorithm for Large-scale set-based G×E tests (SEAGLE) for performing G×E VC tests on biobank data. Here “exact” refers to the fact that SEAGLE computes the original VC test statistic without any approximations, rather than the null distribution of the test statistic being asymptotic or exact. Exactness and scalability are achieved through the judicious use of modern matrix computations, allowing us to dispense with approximations and assumptions. Our numerical experiments illustrate that SEAGLE produces Type 1 error rates and power identical to those of the original GxE VC methods (Tzeng et al., 2011), but at a fraction of the speed. Additionally, SEAGLE can easily handle biobank-scale data with as many as *n* individuals in the order of 10^5^, often at the same speed as state-of-the-art approximate methods (Wang et al., 2020). Compared with the state-of-the-art approximate method (Wang et al., 2020), SEAGLE can produce more accurate Type 1 error rates and power. Another advantage of SEAGLE is its user-friendliness; it can be run on ordinary laptops and does not require specialized or high performance computing equipment or parallelization. In fact, nearly all of our timing comparisons in Section 3 were performed on a 2013 Intel Core i5 laptop with a 2.70 GHz CPU and 16 GB RAM, specs that are standard for modern laptops. Therefore, SEAGLE makes it possible to run exact and scalable G×E VC tests on biobank-scale data with just a modicum of computational resources.

The rest of the paper proceeds as follows. Section 2 describes the standard mixed effects model for G×E effects, testing procedures, computational performance, and SEAGLE algorithm. Section 3 illustrates SEAGLE’s performance through numerical studies. Section 4 concludes with a brief summary of our contributions and avenues for future work.

## 2 Materials and Methods

We describe the standard mixed effects model for G×E effects and testing procedures (Section 2.1), the computational challenges for biobank-scale data (Section 2.2), the components of the SEAGLE algorithm (Section 2.3), and the SEAGLE algorithm as a whole (Section 2.3.4).

### 2.1 G×E Variance Component Tests for Continuous Traits

We present the standard mixed effects model for studying G×E effects, the score-like test statistic, and its *p*-value. Let $y\in R^{n}$ denote the response vector with *n* individual responses for a continuous trait; $X\in R^{n\times p}$ the design matrix of *p* covariates whose leading column is the all ones vector for the intercept; $E\in R^{n}$ the design vector of the environmental factor in the G×E effect; and $G\in R^{n\times L}$ the genetic marker matrix for the *L* SNPs where *L* < *n*. Define the design matrix for the G×E terms as $\tilde{G}=\mathrm{diag}\left(E\right)G\in R^{n\times L}$ where $\mathrm{diag}\left(E\right)\in R^{n\times n}$ is a diagonal matrix with the elements of the vector **E** on the diagonal.

Consider the linear mixed effects model (Tzeng et al., 2011; Lin et al., 2013),

$$y=X\beta_{X}+E\beta_{E}+Gb+\tilde{G}c+\varepsilon .$$ (1)

Here, $\beta_{X}\in R^{p}$ and $\beta_{E}\in R$ are the fixed-effects coefficients for the covariates and environmental factor, respectively; $b\in R^{L}$ and $c\in R^{L}$ are the genetic main (G) effect and G×E effect, respectively, with **b** ∼N(**0**, *τ* **I**_*L*_) and **c** ∼N(**0**, *ν* **I**_*L*_); ***ε*** ∼N(**0**, *σ* **I**_*n*_); and $I_{k}\in R^{k\times k}$ denotes the identity matrix of dimension *k*.

The SNP-set analysis models the G and G×E effects of the *L* SNPs as random effects rather than fixed effects. This choice avoids power loss for non-small *L* and numerical difficulties from correlated SNPs that can occur in a fixed effects model. To assess the presence of G×E effects with *H*_0_ : **c** = **0** in Model (1), one can apply a score-like test to the corresponding variance component with *H*_0_ : *ν* = 0.

To simplify the null model of (Eq. 1) in the score-like test, we consolidate and define $\tilde{X}=\left(\begin{array}{cc} X & E \end{array}\right)\in R^{n\times P}$ and $\beta ={\left(\begin{array}{cc} \beta_{X}^{T} & \beta_{E}^{T} \end{array}\right)}^{T}\in R^{P}$ , where *P* = *p* + 1. The resulting null model becomes $y=\tilde{X}\beta +Gb+\varepsilon$ , where the response is $y∼\text{N}\left(\tilde{X}\beta \;,\;V\right)$ with **V** = *τ* **GG** ^*T*^ + *σ* **I**_*n*_. Following (Tzeng et al., 2011), the score-like test statistic is

$$\begin{array}{ccc} T & = & \frac{1}{2}\;{\left(y-\hat{\mu }\right)}^{T}V^{-1}\tilde{G}{\tilde{G}}^{T}V^{-1}\left(y-\hat{\mu }\right)\\ & = & \frac{1}{2}\;y^{T}P\tilde{G}{\tilde{G}}^{T}Py\equiv \frac{1}{2}t^{T}t,\text{ where }t={\tilde{G}}^{T}Py. \end{array}$$ (2)

In (Eq. 2), $\hat{\mu }=\tilde{X}\hat{\beta }=\tilde{X}{\left({\tilde{X}}^{T}V^{-1}\tilde{X}\right)}^{-1}{\tilde{X}}^{T}V^{-1}y$ and $P=V^{-1}-V^{-1}\tilde{X}{\left({\tilde{X}}^{T}V^{-1}\tilde{X}\right)}^{-1}{\tilde{X}}^{T}V^{-1}$ . Supplementary Appendix S1 presents the restricted maximum likelihood (REML) expectation-maximization (EM) algorithm for estimating the nuisance VC parameters *τ* and *σ* for computing *T* (Tzeng et al., 2011; Zhao et al., 2015). The test statistic *T* follows a weighted $\chi_{\left(1\right)}^{2}$ distribution asymptotically under *H*_0_ : *ν* = 0. That is, $T∼\sum_{\ell }\lambda_{\ell }\chi_{\left(1\right)}^{2}$ , where *λ*_*ℓ*_’s are the eigenvalues of

$$C=C_{1}C_{1}^{T},\;\text{where}\;C_{1}=\frac{1}{\sqrt{2}}V^{\frac{1}{2}}P\tilde{G}.$$ (3)

Given the *λ*_*ℓ*_’s, the *p*-value of *T* can be computed with the moment matching method in Liu et al. (2009) or the exact method in Davies (1980).

### 2.2 Computational Challenges in G×E Variance Component Tests for Biobank-Scale Data

We identify three computational bottlenecks.

1. The test statistic *T* and the p-value computation depend on $P\in R^{n\times n}$ , which in turn depends on $V^{-1}\in R^{n\times n}$ . Explicit formation of the inverse is too expensive and numerically inadvisable, due to loss of numerical accuracy and stability (Higham, 2002, Chapter 14).
2. The REML EM algorithm (Supplementary Appendix S1) estimates the nuisance variance components *τ* and *σ* in **V** under the null hypothesis. Each iteration requires products with the orthogonal projector $I-\tilde{X}{\left({\tilde{X}}^{T}\tilde{X}\right)}^{-1}{\tilde{X}}^{T}$ , and inverting a matrix of dimension *n* − *P* ≈ *n*.
3. Computing the p-values requires two eigenvalue decompositions: 1) an eigenvalue decomposition of **V** to compute $V^{\frac{1}{2}}$ in **C**_1_; and 2) an eigenvalue decomposition of **C** to compute the *λ*_*ℓ*_’s in the weighted $\chi_{\left(1\right)}^{2}$ distribution. Computing the eigenvalues and eigenvectors of the symmetric matrix $V\in R^{n\times n}$ requires $O\left(n^{3}\right)$ arithmetic operations and $O\left(n^{3}\right)$ storage. Computing the eigenvalues of $C\in R^{n\times n}$ requires another $O\left(n^{3}\right)$ arithmetic operations.

### 2.3 Components of the SEAGLE Algorithm for Biobank-Scale GxE Variance Component Test

We present our approach for overcoming the three computational challenges in the previous section: Multiplication with **V** ^−1^ without explicit formation of the inverse (Section 2.3.1), a scalable REML EM algorithm (Section 2.3.2), and a scalable algorithm for computing the eigenvalues of **C** (Section 2.3.3). The idea is to replace explicit formation of inverses by low-rank updates and linear system solutions; and to replace *n* × *n* eigenvalue decompositions with *L* × *L* ones.

#### 2.3.1 Multiplication by **V** ^−1^ Without Explicit Formation of **V** ^−1^

The test statistic *T* and its *p*-value calculation depend on **V** ^−1^. We avoid the explicit formation of the inverse by viewing **V** = *τ* **GG** ^*T*^ +*σ* **I**_*n*_ as the low-rank update of a diagonal matrix, and then applying the Sherman-Morrison-Woodbury formula below to reduce the dimension of the computed inverse from *n* to *L* where *L* ≪ *n*.

LEMMA 1 [Section 2.1.4 in Golub and Van Loan (2013)]. Let $H\in R^{n\times n}$ be nonsingular, and let $U,B\in R^{n\times L}$ so that **I** + **B** ^*T*^ **H** ^−1^ **U** is nonsingular. Then

$${\left(H+UB^{T}\right)}^{-1}=H^{-1}-H^{-1}U{\left(I+B^{T}H^{-1}U\right)}^{-1}B^{T}H^{-1}.$$

Applying Lemma 1 to the product of the inverse of $V=\sigma \left(I_{n}+\frac{\tau }{\sigma }GG^{T}\right)$ with any right-hand side input $W\in R^{n\times l}$ gives

$$V^{-1}W=\frac{1}{\sigma }\left[W-\frac{\tau }{\sigma }G{\left(I_{L}+\frac{\tau }{\sigma }G^{T}G\right)}^{-1}G^{T}W\right],$$ (4)

which reduces the dimension of the inverse from *n* to *L*. The explicit computation of the inverse of $M=I_{L}+\frac{\tau }{\sigma }G^{T}G\in R^{L\times L}$ is, in turn, avoided with a Cholesky decomposition followed by a linear system solution. Algorithm 1 shows pseudocode for computing (Eq. 4). As a further saving, we pre-compute the Cholesky factorization of **M** only once, so it is available for re-use in the computation of the test statistic and *p*-value.

**Algorithm 1:** applyVinv

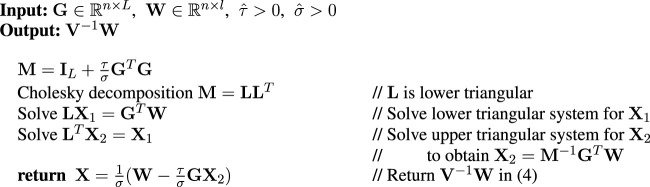

#### 2.3.2 Scalable Restricted Maximum Likelihood Expectation-Maximization Algorithm

We present the scalable version of the REML EM algorithm in Supplementary Appendix S1 that avoids explicit formation of the orthogonal projector and the inverses.

We assume throughout that $\tilde{X}$ has full column rank with $rank\left(\tilde{X}\right)=P$ , and let $range\left(\tilde{X}\right)$ be the space spanned by the columns of $\tilde{X}$ . The space perpendicular to $range\left(\tilde{X}\right)$ is $range{\left(\tilde{X}\right)}^{⊥}$ , and the orthogonal projector onto this space is

$$AA^{T}=I-\tilde{X}{\left({\tilde{X}}^{T}\tilde{X}\right)}^{-1}{\tilde{X}}^{T}$$ (5)

where $A\in R^{n\times \left(n-P\right)}$ has orthonormal columns with **A** ^*T*^ **A** = **I**_*n*−*P*_. Let $u=A^{T}y\in R^{n-P}$ be the orthogonal projection of the response onto $range{\left(\tilde{X}\right)}^{⊥}$ .

In iteration *t* + 1 of the algorithm, define

$$\hat{R}={\hat{\tau }}_{t}\;A^{T}GG^{T}A+{\hat{\sigma }}_{t}\;I_{n-P}\in R^{\left(n-P\right)\times \left(n-P\right)}.$$ (6)

Then the updates in Supplementary Eq. S5 and Supplementary Eq. S6 from Supplementary Appendix S1 can be expressed as

$$\begin{array}{ccc} {\hat{\tau }}_{t+1} & = & \frac{{\hat{\tau }}_{t}}{L}\left[{\hat{\tau }}_{t}\;\|G^{T}A{\hat{R}}^{-1}u{\|}_{2}^{2}+\text{trace}\left(I_{L}-{\hat{\tau }}_{t}G^{T}A{\hat{R}}^{-1}A^{T}G\right)\right]\\ {\hat{\sigma }}_{t+1} & = & \frac{{\hat{\sigma }}_{t}}{n-P}\left[\|{\hat{R}}^{-1}u{\|}_{2}^{2}+{\hat{\tau }}_{t}\;trace\left(G^{T}A{\hat{R}}^{-1}A^{T}G\right)\right]. \end{array}$$

The two bottlenecks in the REML EM algorithm are the computation of the non-symmetric “square-root” **A** in (Eq. 5), and products with ${\hat{R}}^{-1}$ from (Eq. 6). Since *P* is small, *n* − *P* ≈ *n*, hence explicit formation of the inverse is out of the question, especially since $\hat{R}$ changes in each iteration due to the updates for ${\hat{\tau }}_{t}$ and ${\hat{\sigma }}_{t}$ .

To avoid explicit formation of the full matrix in (Eq. 5), we compute instead the QR decomposition

$$\tilde{X}=\underset{Q}{\underset{︸}{\left(\begin{array}{cc} Q_{1} & Q_{2} \end{array}\right)}}\left(\begin{array}{c} R_{0}\\ 0 \end{array}\right),$$ (7)

where $Q\in R^{n\times n}$ is an orthogonal matrix with **Q** ^*T*^ **Q** = **QQ** ^*T*^ = **I**_*n*_. The columns of $Q_{1}\in R^{n\times P}$ form an orthonormal basis for $range\left(\tilde{X}\right)$ , and the columns of $Q_{2}\in R^{n\times \left(n-P\right)}$ form an orthonormal basis for $range{\left(\tilde{X}\right)}^{⊥}$ . The upper triangular matrix $R_{0}\in R^{P\times P}$ is nonsingular, due to the assumption of $\tilde{X}$ having full column rank. Therefore, (Eq. 5) simplifies to

$$I-\tilde{X}{\left({\tilde{X}}^{T}\tilde{X}\right)}^{-1}{\tilde{X}}^{T}=I-Q_{1}Q_{1}^{T}=Q_{2}Q_{2}^{T}.$$

Thus, **A** = **Q**_2_ represents the trailing *n* − *P* columns of the orthogonal matrix **Q** in the QR factorization of $\tilde{X}$ .

Algorithm 2 shows pseudocode for the REML EM algorithm. Since **A** = **Q**_2_ occurs only as **A** ^*T*^ in matrix-vector or matrix-matrix multiplications, we do not compute **A** explicitly. Instead, we compute the full QR decomposition in (Eq. 7) where the “QR object” $Q=\left(\begin{array}{cc} Q_{1} & A \end{array}\right)$ is stored implicitly in factored form. To compute **u** = **A** ^*T*^ **y**, we multiply $\tilde{u}=Q^{T}y=\left(\begin{array}{c} Q_{1}^{T}y\\ A^{T}y \end{array}\right)$ and then extract the trailing *n* − *P* rows from $\tilde{u}$ .

**Algorithm 2:** REML-EM.

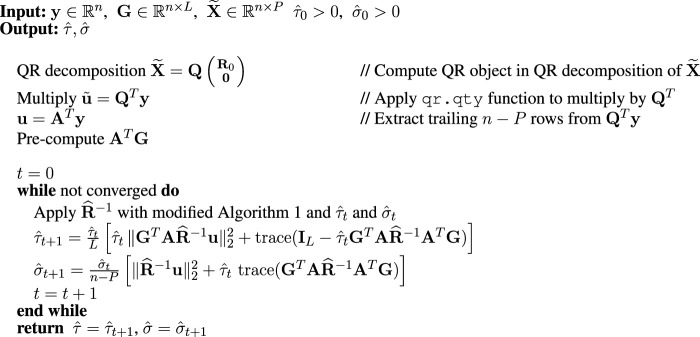

Furthermore, we apply ${\hat{R}}^{-1}$ from (Eq. 6) with a modified version of Algorithm 1 where **G** is replaced by **A** ^*T*^ **G** and **I**_*n*_ by **I**_*n*−*P*_. Unfortunately, one cannot pre-compute a Cholesky factorization for the whole algorithm since ${\hat{\tau }}_{t}$ and ${\hat{\sigma }}_{t}$ change in each iteration. However, within a single iteration, we pre-compute a Cholesky factorization of $\hat{R}$ for subsequent linear system solutions of ${\hat{R}}^{-1}u$ and ${\hat{R}}^{-1}A^{T}G$ . Following previous work on G×E VC tests in Tzeng et al. (2011), our convergence criteria are: i) the magnitude of the relative difference between the current and previous estimate; and ii) the default convergence tolerance from the SIMreg package for R.

#### 2.3.3 Scalable Algorithm for Computing the Eigenvalues of C

Computation of the *p*-values requires the eigenvalues of $C=C_{1}C_{1}^{T}$ in (Eq. 3), which in turn involves products with $V^{\frac{1}{2}}\in R^{n\times n}$ . We avoid the computation of the square root by exploiting the fact that the nonzero eigenvalues of $C_{1}C_{1}^{T}$ are equal to the nonzero eigenvalues of $C_{1}^{T}C_{1}$ . The symmetry of **V** and **P** and the equality **PVP** = **P** imply the much simpler expression

$$C_{1}^{T}C_{1}=\frac{1}{2}{\tilde{G}}^{T}PVP\tilde{G}=\frac{1}{2}{\tilde{G}}^{T}P\tilde{G}.$$

The explicit formation of **P** is avoided by computing instead products $P\tilde{G}$ with Algorithm 1. Therefore, our approach of replacing the *n* × *n* matrix $C_{1}C_{1}^{T}$ with the much smaller *L* × *L* matrix $C_{1}^{T}C_{1}$ reduces the operation count from $O\left(n^{3}\right)$ down to $O\left(L^{3}\right)$ . Part III of Algorithm 3 shows the pseudocode.

**Algorithm 3:** SEAGLE.

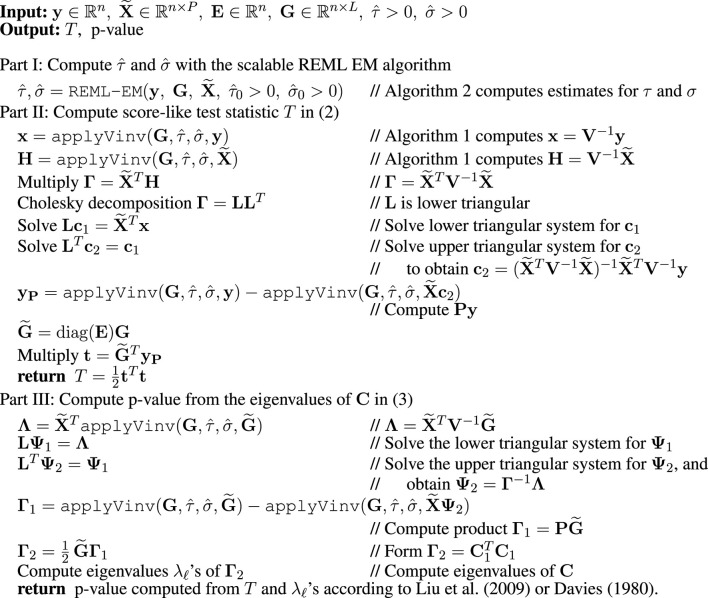

#### 2.3.4 The SEAGLE Algorithm

Combining the algorithms from Section 2.3.1, Section 2.3.2, and Section 2.3.3 gives the SEAGLE Algorithm 3 for computing the score-like test statistic *T* and its *p*-value. SEAGLE is implemented in the publicly available R package SEAGLE.

Algorithm 3 consists of three parts. Part I computes $\hat{\tau }$ and $\hat{\sigma }$ with the scalable REML EM in Algorithm 2; Part II computes the score-like *T* statistic in (Eq. 2); and Part III computes the *p*-values from the eigenvalues of **C** in (Eq. 3). Linear systems with **V** are efficiently solved with Algorithm 1. The fast diagonal multiplication in R stores diagonal matrices as vectors. The QR decomposition is implemented with the qr function in the R base package. The qr.qty function makes it possible to left multiply by **Q** ^*T*^ without having to explicitly form **Q**.

## 3 Results

### 3.1 Simulation Study

We evaluate the performance of our proposed method SEAGLE using simulation studies from two settings. In the first, we simulate data from a random effects genetic model according to Model (1). This enables us to evaluate SEAGLE’s estimation and testing performance. We include experiments with a smaller *n* = 5,000 to enable comparisons with existing G×E VC tests as well as larger *n* values (i.e., 20,000 and 100,000) to demonstrate SEAGLE’s effectiveness on biobank-scale data. In the second, we simulate data from a fixed effects genetic model with larger *n* = 20,000 and *n* = 100,000 observations. This enables us to evaluate the testing performance when the data do not follow our modeling assumptions.

In each setting, we study the Type 1 error rate and power. We consider three baseline approaches: i) the original G×E VC test (referred to as OVC) (Tzeng et al., 2011; Wang et al., 2015b), as implemented in the SIMreg R package (https://www4.stat.ncsu.edu/˜jytzeng/software_simreg.php); ii) FastKM (Marceau et al., 2015), as implemented in the FastKM R package; and iii) MAGEE (Wang et al., 2020), as implemented in the MAGEE R package. MAGEE is the state-of-the-art scalable G×E VC test with demonstrated superior performance compared to several set-based GxE methods.

In all simulations, we obtain the genotype design matrix $G\in R^{n\times L}$ as follows. First, we employ the COSI software (Schaffner et al., 2005) to simulate 10,000 haplotypes of SNP sequences mimicking the European population. We then form a SNP set of *L* loci (*L* = 100 or 400) with minor allele frequency (MAF) less than 1% by randomly selecting *L* SNPs without replacement. Finally, in each replicate, we generate the genotypes of *n* individuals by randomly selecting two haploytpes with replacement. We also consider a confounding factor $X\in R^{n}$ and an environmental factor $E\in R^{n}$ , where each is generated from a standard normal distribution. Given *X* and *E*, we then form the covariate design matrix $\tilde{X}\in R^{n\times 3}$ by column-combining the vector of ones, *X*, and *E* together.

#### 3.1.1 Random Effects Simulation Study

Given the *n* × *L* genotype design matrix **G** (where *L* = 100 for *n* = 5, 20, and 100 k, and *L* = 400 for *n* = 20 and 100 k) and the *n* × *P* covariate design matrix $\tilde{X}$ (where *P* = 3), we simulate the outcome data **y** according to the random effects model: $y=\tilde{X}\beta +Gb+\mathrm{diag}\left(E\right)Gc+e$ , where ***β*** is set as the all ones vector of length *P*; **b** is generated from N(**0**, *τ* **I**_*L*_); **e** is generated from N(**0**, *σ* **I**_*n*_); and *σ* and *τ* are set to be 1. We set *ν* = 0 for Type I error analysis and *ν* > 0 for power analysis, where the actual value of *ν* is set so that the empirical power of various methods can be within 0.2 ∼0.9 when possible (i.e., not all near 1 or all < 0.1) and would depend on sample size (i.e., *ν* = 0.04, 0.002 and 0.007 for *n* = 5, 20, and 100 k, respectively). With *ν* = 0, we simulate *N* = 1,000 replicates and evaluate the results at the nominal level *α* = 0.05, except when assessing SEAGLE’s Type I error rates at *α* = 5 × 10^−2^, 5 × 10^−3^, 5 × 10^−4^, 5 × 10^−5^, and 2.5 × 10^−6^, where we consider *N* = 20,000,000 replicates. With *ν* > 0, we simulate *N* = 200 replicates to assess power.

**FIGURE 1:**
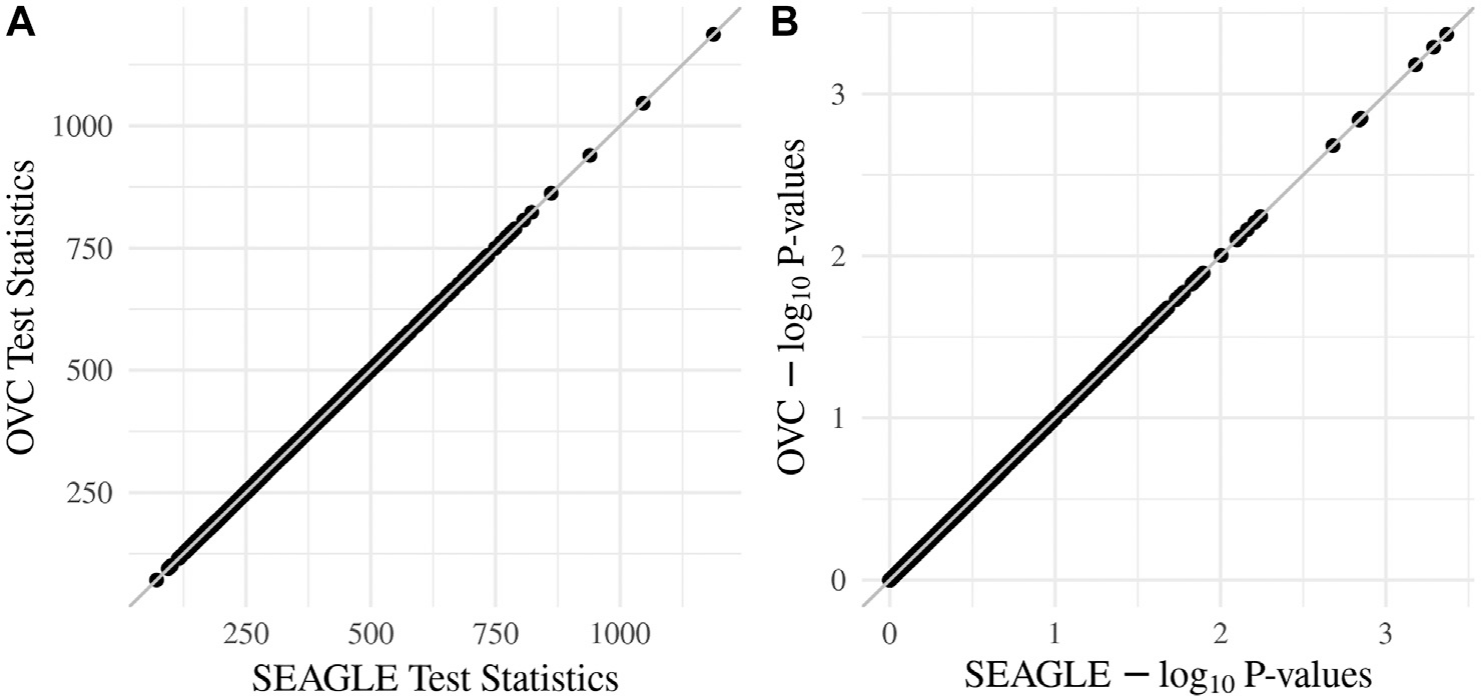
SEAGLE vs. original G×E VC (OVC) results based on *N* = 1, 000 replicates for *n* = 5,000 observations and *L* = 100 loci under *H*_0_: no G×E effect (*ν* = 0). The **(A,B)** show the scatter plots of the testing results computed from SEAGLE vs. those from OVC, depicting the “exact” relationship between SEAGLE and OVC; **(A)** depicts test statistics *T* and **(B)** depicts the p-values.

We begin by examining the Type 1 error rate for SEAGLE with *N* = 20,000,000 replicates. Table 1 depicts the SEAGLE Type 1 error rates and shows that SEAGLE provides reasonable control over the Type 1 error rate at varying *α*-levels with *p*-values from Davies (1980). Next, under *H*_0_: *ν* = 0 and *τ* = *σ* = 1 with *N* = 1,000 replicates, we compare the testing results of SEAGLE with OVC. Table 2 shows the bias and the mean square error (MSE) of the estimated values for *τ* and *σ* obtained from the SEAGLE and OVC REML EM algorithms. Both algorithms produce very small bias and MSE for *τ* and *σ*. The left and right panels in Figure 1 depicts scatter plots of the score-like test statistics and *p*-values, respectively, produced by SEAGLE and OVC. The panels show that SEAGLE and OVC produce identical test statistics and *p*-values, hence the “exactness” of the SEAGLE algorithm.

**TABLE 1:** Type 1 error rate with 95*%* confidence intervals (CIs) for SEAGLE with Davies *p*-values over *N* = 20,000,000 replicates for *n* = 5,000 observations, *L* = 100 loci, and variance components *τ* = *σ* = 1 and *ν* = 0.

*α*-level	Type 1 error	Std. Error	95% CI
5 × 10^−2^	0.0497635	0.0000486	(0.0496681, 0.0498588)
5 × 10^−3^	0.0049571	0.0000157	(0.0049263, 0.0049878)
5 × 10^−4^	0.0004894	0.0000049	(0.0004797, 0.0004990)
5 × 10^−5^	0.0000481	0.0000016	(0.0000451, 0.0000511)
2.5 × 10^−6^	0.0000027	0.0000004	(0.0000020, 0.0000035)

**TABLE 2:** SEAGLE vs. original G×E VC (OVC) results based on *N* = 1,000 replicates for *n* = 5,000 observations and *L* = 100 loci under *H*_0_: no G×E effect (*ν* = 0). Table shows the bias and mean square error (MSE) of the estimated *τ* and *σ* values, compared to the true values *τ* = *σ* = 1.

		*τ*	*σ*
SEAGLE	Bias	−3.06 × 10^−4^	−1.01 × 10^−3^
	MSE	4.37 × 10^−2^	4.18 × 10^−4^
OVC	Bias	−6.93 × 10^−4^	−5.56 × 10^−4^
	MSE	4.36 × 10^−2^	1.05 × 10^−4^

Since the data are generated from a random effects model under *H*_0_: *ν* = 0, we can compute the “true” score-like test statistic *T* by evaluating (Eq. 2) at the true *τ* and *σ* values, and obtaining the corresponding *p*-value. We refer to this as the “Truth” and include it as a baseline approach. Supplementary Figure S1 depicts quantile-quantile plots (QQ plots) of the *p*-values from different methods, each over *N* = 1,000 replicates. We use the Kolmogorov-Smirnov (KS) test to examine whether or not these observed *p*-values follow the expected null distribution, i.e., to test for *H*_0_: the observed *p*-values follows Uniform (0,1). With *τ* = *σ* = 1, all methods exhibit similar *p*-value behavior and follow Uniform (0,1) except MAGEE (Supplementary Figure S1A). In fact, the points for SEAGLE (red) and OVC (light blue) overlap. The corresponding *p*-values of the KS test are 0.3599, 0.4303, 0.4303, 0.3529, and 3.33e-15 for Truth, SEAGLE, OVC, FastKM, and MAGEE, respectively. The deviation of MAGEE’s *p*-value distribution is likely due to the non-negligible genetic main effects as reported in Wang et al. (2020). To confirm, we repeat the same simulation with *τ* = 0.01 and *σ* = 1 and examine the *p*-values of MAGEE. The results based on *N* = 1,000 replicates show that the MAGEE *p*-values behavior similarly to Uniform (0,1) (Supplementary Figure S1B). The *p*-values of the KS test are 0.7244, 0.4945, and 0.1426 for Truth, SEAGLE and MAGEE, respectively.

In Table 3, we compute the MSE of the *p*-values obtained from SEAGLE, OVC, FastKM, and MAGEE, compared to the Truth *p*-values at *τ* = *σ* = 1. We observe that MAGEE produces *p*-values with larger MSE than the other methods. Supplementary Figure S2 shows the corresponding absolute relative error of the *p*-values for each method, computed by first taking the absolute difference between a method’s *p*-value and the Truth *p*-value, then dividing it by the Truth *p*-value. The boxplots suggest that MAGEE exhibits higher bias and greater variance than SEAGLE, OVC and FastKM at *τ* = *σ* = 1.

**TABLE 3:** Mean squared error (MSE) of the p-values obtained from different G×E VC tests, compared to the “Truth” p-values. Results are obtained with *τ* = *σ* = 1 under *H*_0_: *ν* = 0 over *N* = 1,000 replicates with *n* = 5,000 observations and *L* = 100 loci.

	MSE of *p*-value
SEAGLE	3.49 × 10^−4^
MAGEE	224.96 × 10^−4^
OVC	3.49 × 10^−4^
FastKM	17.88 × 10^−4^

**FIGURE 2:**
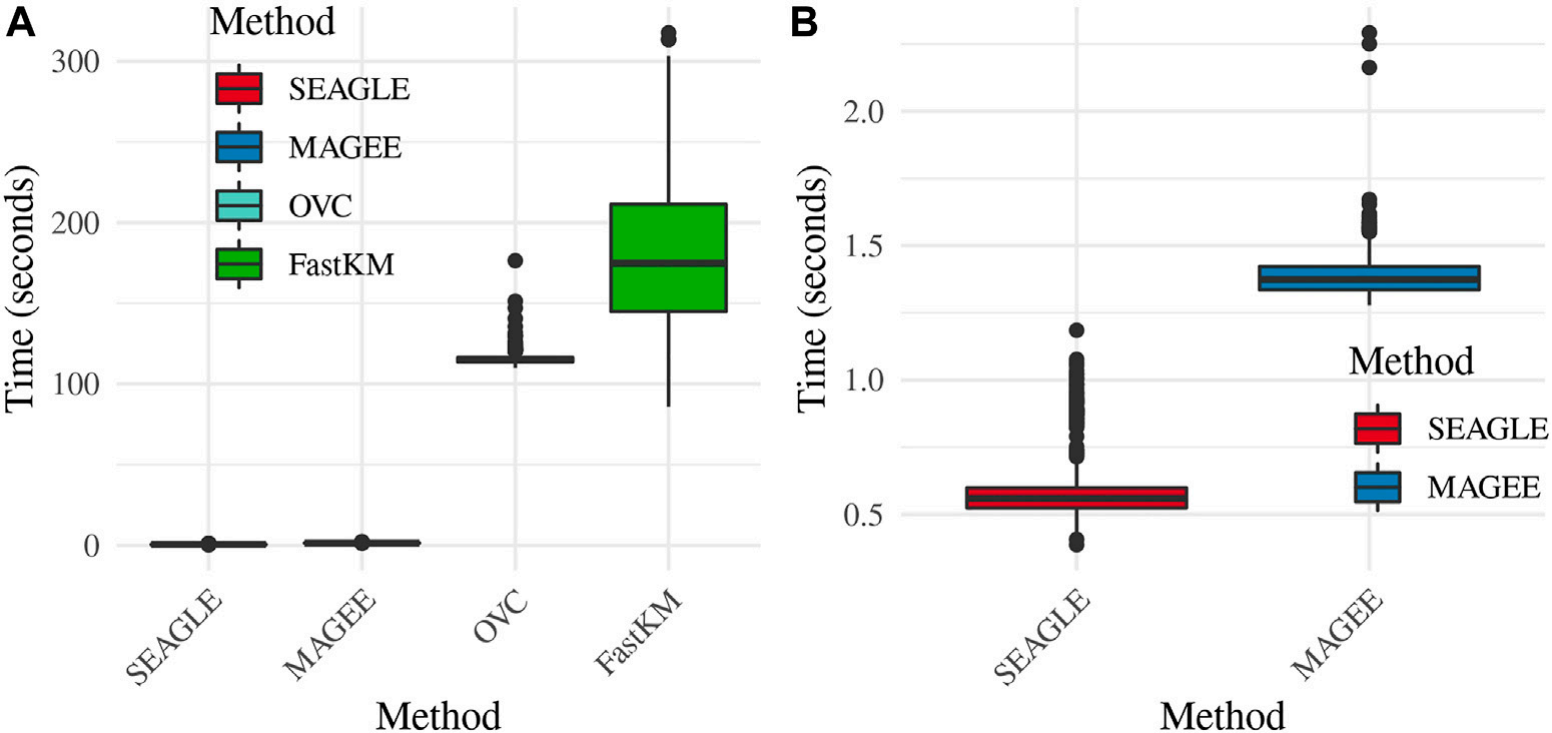
**(A)** Box plots of computation time in seconds to obtain a single *p*-value over *N* = 1,000 replicates for *n* = 5,000 observations and *L* = 100 loci. **(B)** Computation time in seconds to obtain a single *p*-value for SEAGLE and MAGEE.

Regarding the computational cost, the left panel in Figure 2 shows boxplots of the computation time in seconds required to obtain a single *p*-value for each of the methods over *N* = 1,000 replicates with *τ* = *σ* = 1 and *ν* = 0. The right panel shows the same boxplots for just SEAGLE and MAGEE. Results show that at *n* = 5,000 observations and *L* = 100 loci, SEAGLE is faster than MAGEE on average. All replicates were computed on a 2013 Intel Core i5 laptop with a 2.70 GHz CPU and 16 GB RAM.

**FIGURE 3:**
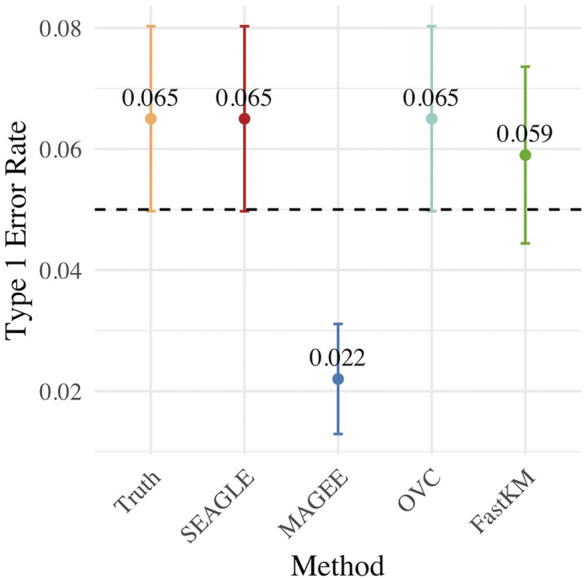
Type 1 error at *α* = 0.05 level for *N* = 1,000 replicates with *n* = 5,000 observations and *L* = 100 loci with *τ* = *σ* = 1 and *ν* = 0.

Figure 3 shows the Type 1 error rate for each method under *H*_0_: *ν* = 0. SEAGLE performs identically to OVC with respect to Type 1 error rate at *α* = 0.05 while requiring only a fraction of the computation time, as demonstrated in Figure 2. By contrast, MAGEE is nearly as fast as SEAGLE for *n* = 5,000 and *L* = 100 but produces much more conservative *p*-values at *τ* = *σ* = 1.

**FIGURE 4:**
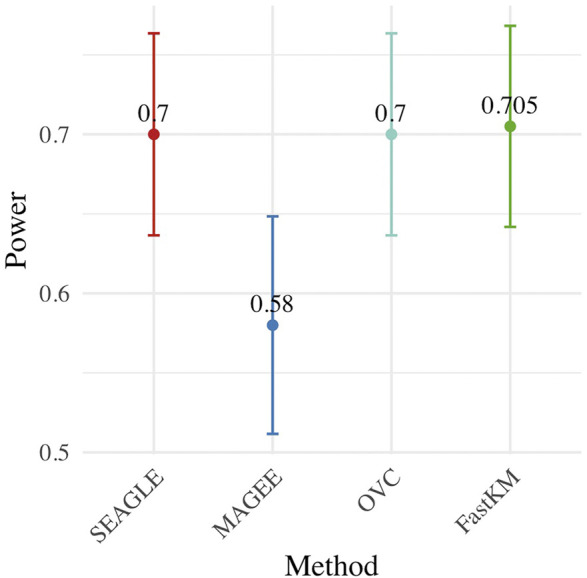
Power at *α* = 0.05 level over *N* = 200 replicates with *n* = 5,000 observations, *L* = 100 loci, and *ν* = 0.04.

Figure 4 shows the power for each method under the alternative hypothesis that *ν* > 0. SEAGLE again performs identically to OVC while requiring a fraction of the computation time. By contrast, MAGEE is nearly as fast as SEAGLE for *n* = 5,000 and *L* = 100 but has lower power when *τ* = *σ* = 1 and *ν* = 0.04.

**FIGURE 5:**
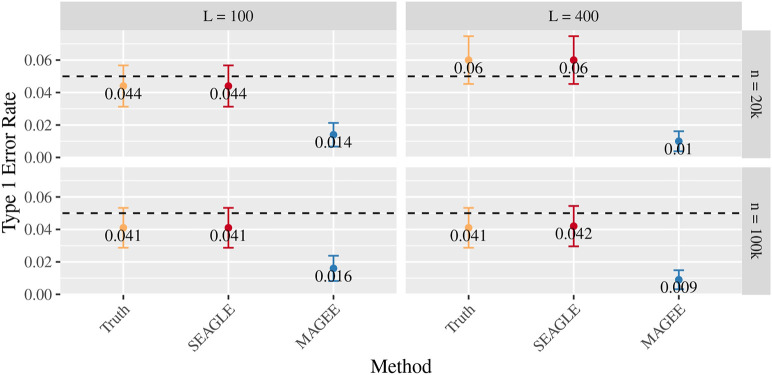
Type 1 error at *α* = 0.05 for random effects simulations with *N* = 1,000 replicates for *n* = 20,000 and *n* = 100,000 observations, *L* = 100 and *L* = 400 loci, and *τ* = *σ* = 1 and *ν* = 0.

**FIGURE 6:**
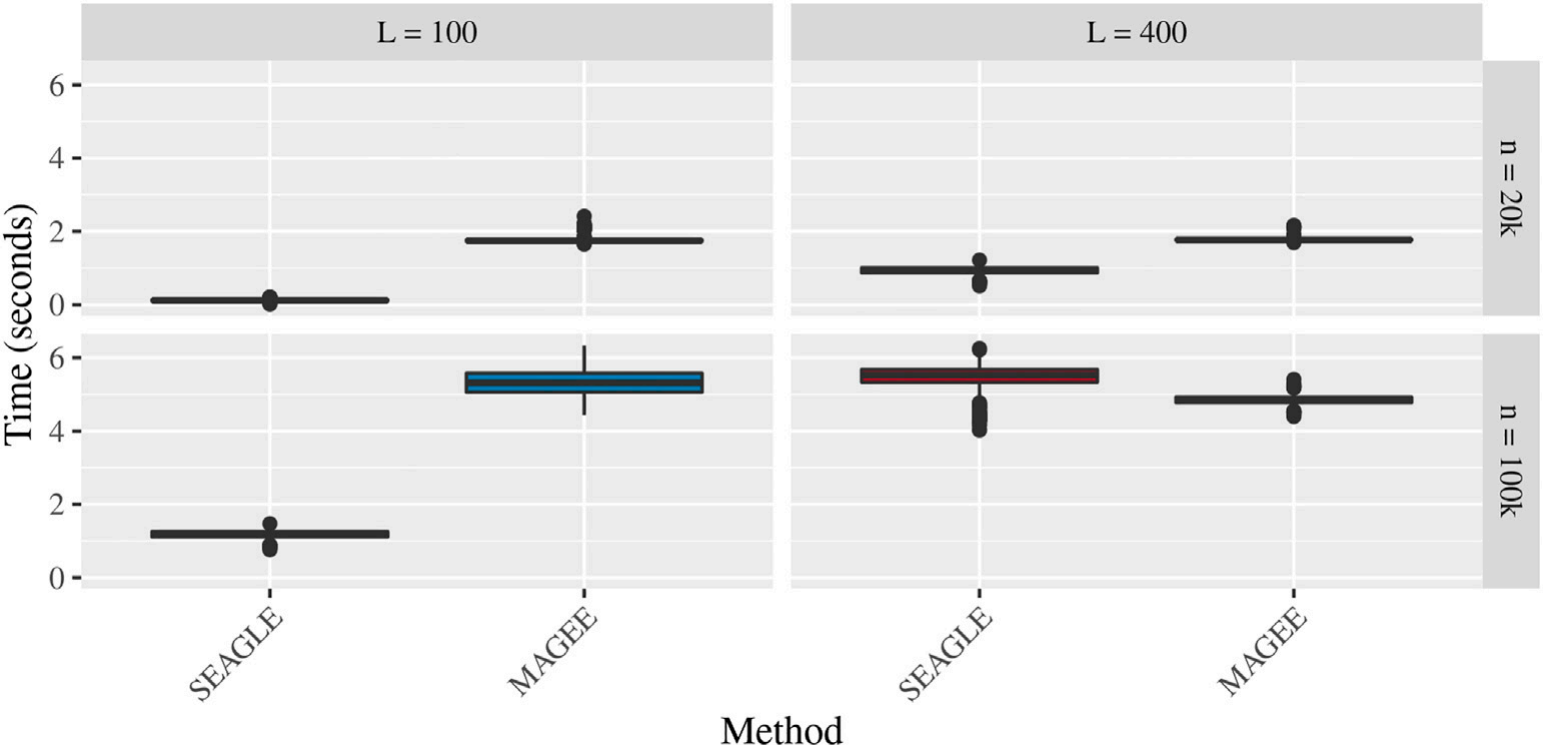
Time in seconds for random effects simulations with *N* = 1,000 replicates for *n* = 20,000 and *n* = 100,000 observations, *L* = 100 and *L* = 400 loci, and *τ* = *σ* = 1 and *ν* = 0. Replicates computed on a single core of an Intel Xeon Gold 6226R (2.90 GHz) machine with 8 GB of RAM.

**FIGURE 7:**
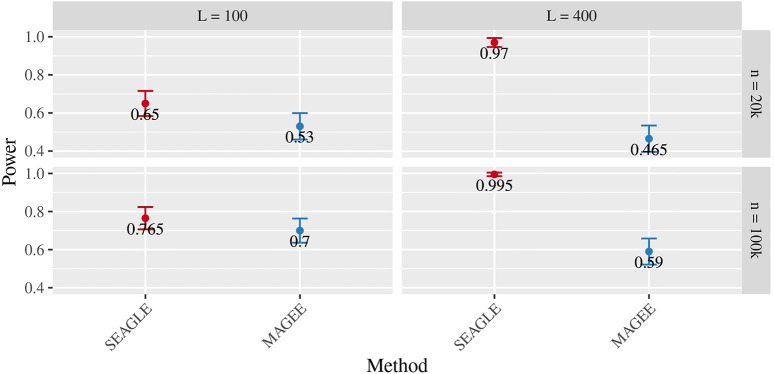
Power at *α* = 0.05 level over *N* = 200 replicates with *n* = 20,000 (*ν* = 0.007) and *n* = 100,000 (*ν* = 0.002) observations, *L* = 100 and *L* = 400 loci, and *τ* = *σ* = 1.

Figures 5–7 show the comparisons of SEAGLE and MAGEE under the large-*n* simulations (i.e., *n* = 20,000 and *n* = 100,000 individuals) with *L* = 100 and *L* = 400 loci and by setting *τ* = *σ* = 1. Figure 5 shows the Type 1 error rate for Truth, SEAGLE, and MAGEE under *H*_0_ : *ν* = 0. SEAGLE performs almost identically to Truth and OVC, and provides adequate coverage at the *α* = 0.05 level. Meanwhile, MAGEE produces conservative *p*-values. Figure 6 depicts boxplots of the computation time in seconds required to obtain a single *p*-value for SEAGLE and MAGEE under *H*_0_ : *ν* = 0. SEAGLE is faster than MAGEE at both *n* = 20,000 and *n* = 100,000 when *L* = 100 and requires comparable computation time to MAGEE when *L* = 400. These timing results were obtained on a single core of an Intel Xeon Gold 6226 R (2.90 GHz) machine with 8 GB of RAM. Figure 7 shows the power for SEAGLE and MAGEE under *H*_*A*_: *ν* > 0 (i.e., *ν* = 0.007 for *n* = 20,000 and *ν* = 0.002 for *n* = 100,000). SEAGLE exhibits greater power than MAGEE in all four scenarios considered.

#### 3.1.2 Fixed Effects Simulation Study

To study the performance of our proposed method when the data may not adhere to our model assumptions, we follow previous work (Marceau et al., 2015; Wang et al., 2020) and simulate data according to the fixed effects model with a given $\tilde{X}$ and **G**:

$$y=\tilde{X}\gamma_{\tilde{X}}+G\gamma_{G}+\mathrm{diag}\left(E\right)G\gamma_{GE}+e,$$ (8)

where $\gamma_{\tilde{X}}$ is the all ones vector of length *P* = 3, $\gamma_{G}\in R^{L}$ , $\gamma_{GE}\in R^{L}$ , and **e** ∼N(**0**,*σ* **I**_*n*_) with *σ* = 1. The entries of ***γ***_*G*_ and ***γ***_*GE*_ pertaining to causal loci are set to be *γ*_*G*_ and *γ*_*GE*_, respectively. The remaining entries of ***γ***_*G*_ and ***γ***_*GE*_ pertaining to non-causal loci are 0. We consider *n* = 20,000 or 100,000 observations with *L* = 100 or 400, and compare SEAGLE with MAGEE only since OVC and fastKM are unable to work on the sample sizes considered here. We select the first *ℓ* loci to be causal (i.e., loci with both G and G×E effects or just G effect). We vary *γ*_*G*_ over 0.5, 1, and 1.5 for the *ℓ* loci to study the impact of the G main effect sizes. For each scenario, we report the signal-to-noise ratio (SNR), obtained by the ratio of the effect-term variance (i.e., **G*γ***_*G*_ or   diag(**E**)**G*γ***_*GE*_) to the error-term variance (i.e., **e**) in Eq. 8. Because each simulation replicate has its own **G**, we report the median variance ratio as SNR.

**FIGURE 8:**
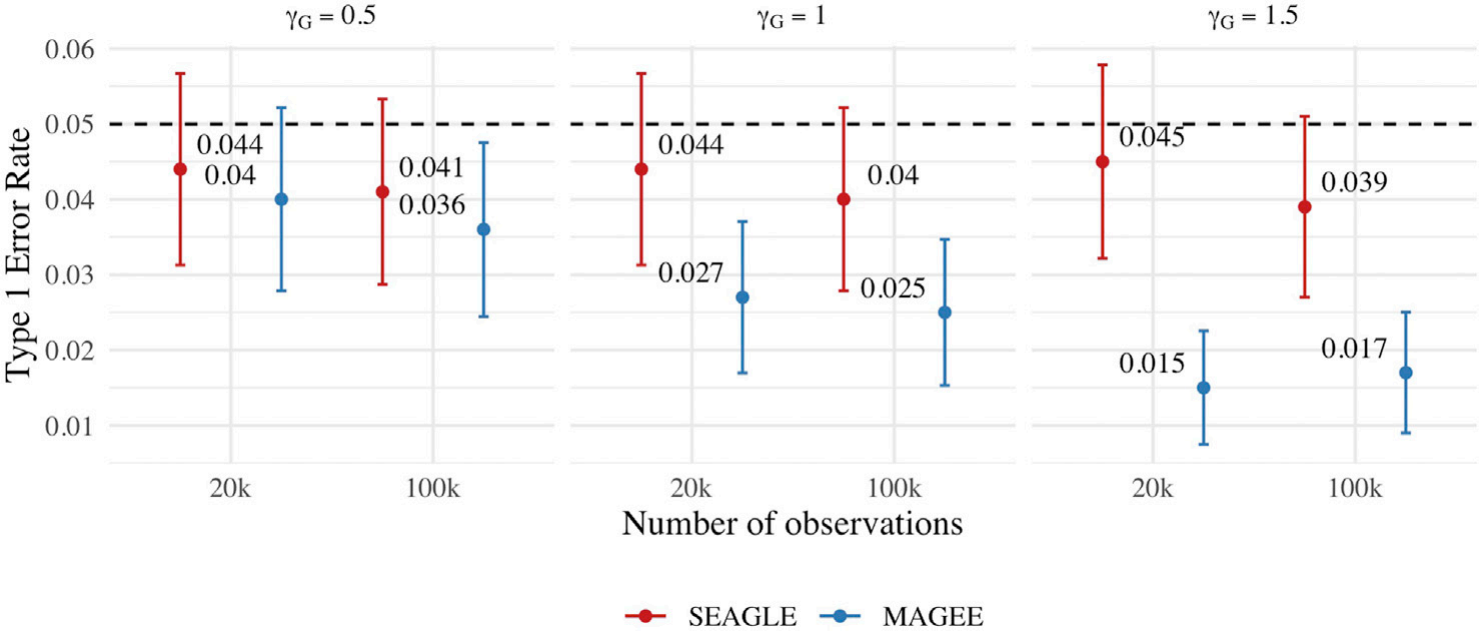
Type 1 error at *α* = 0.05 for fixed effects simulations with *N* = 1,000 replicates with *n* = 20,000 and *n* = 100,000 observations, and *L* = 100 loci with *γ*_*GE*_ = 0 and varying values of *γ*_*G*_.

We first evaluate the Type 1 error of SEAGLE by simulating *N* = 1,000 replicates with *L* = 100 for both *n* = 20,000 and 100,000, and setting *γ*_*GE*_ = 0 for all loci while letting the first *ℓ* = 40 loci to have non-zero *γ*_*G*_. For both sample sizes, the SNR based on   Var(**G*γ***_*G*_)/Var(**e**) of Eq. 8 is 0.015, 0.061 and 0.138 for ***γ***_*G*_ = 0.5, ***γ***_*G*_ = 1 and ***γ***_*G*_ = 1.5, respectively. Figure 8 depicts the Type 1 error rate at *α* = 0.05 over varying values for *γ*_*G*_. While the Type 1 error rate for SEAGLE remains relatively unaffected by different *γ*_*G*_ values, MAGEE produces more conservative *p*-values as *γ*_*G*_ increases. This is consistent with the MAGEE assumption requiring a small G main effect (Wang et al., 2020). Supplementary Figure S3 shows the corresponding quantile-quantile plots for the *p*-values obtained from SEAGLE and MAGEE.

**FIGURE 9:**
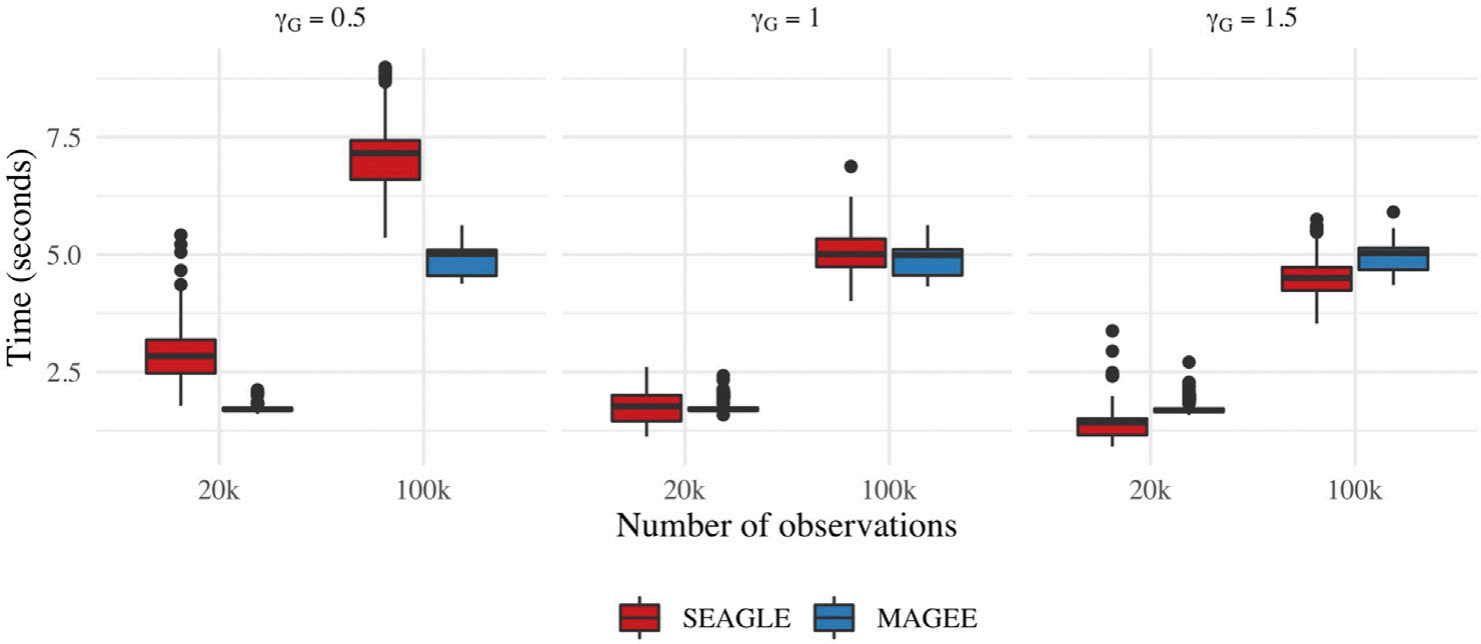
Computation time in seconds for fixed effects simulations with *N* = 1,000 replicates with *n* = 20,000 observations and *L* = 100 loci.

Figure 9 depicts boxplots of the computation time in seconds required to obtain a single *p*-value over the *N* = 1,000 replicates for *n* = 20,000 and *n* = 100,000, and *L* = 100, over varying values for *γ*_*G*_. All replicates were computed on a 2013 Intel Core i5 laptop with a 2.70 GHz CPU and 16 GB RAM. For *n* = 20,000, SEAGLE is faster than MAGEE at larger values of *γ*_*G*_ even though MAGEE computes an approximation to the test statistic *T* and bypasses the traditional REML EM algorithm. At smaller values of *γ*_*G*_, however, SEAGLE requires a few seconds more than MAGEE. This is because smaller *γ*_*G*_ values result in smaller *τ*, and the REML EM algorithm converges slowly for *τ* values close to 0. Supplementary Figure S4 illustrates this empirically for *n* = 20,000 with the estimated values of *τ* produced by the REML EM algorithm at different *γ*_*G*_ values. These trends persist for *n* = 100,000 observations.

For power evaluation, we simulate *N* = 200 replicates with *L* = 100 and 400, and let the first *ℓ* causal loci to have non-zero *γ*_*G*_ and *γ*_*GE*_. For *L* = 100 loci, we set *γ*_*GE*_ for the first *ℓ* = 40 causal loci to be 0.1 or 0.15. For *L* = 400 loci, we set *γ*_*GE*_ for the first *ℓ* = 120 causal loci to be 0.075 or 0.1. These *γ*_*GE*_ values are determined so that the power for *n* = 20,000 at *α* = 0.05 is not close to 1. When *L* = 100, the SNR for the G×E effect based on Var(diag(**E**)**G*γ***_*GE*_)/Var(**e**) of (Eq. 8) is 0.0006 and 0.0015 for ***γ***_*GE*_ = 0.1 and 0.15, respectively. When *L* = 400, the SNR for the GxE effect is 0.0014 and 0.0024 for ***γ***_*GE*_ = 0.075 and 0.1, respectively. The values of *γ*_*G*_ for the *ℓ* causal loci are set to be 0.5, 1.0, and 1.5 as before. For *L* = 100, the SNR for the G main effect is 0.015, 0.061 and 0.138 for ***γ***_*G*_ = 0.5, ***γ***_*G*_ = 1 and ***γ***_*G*_ = 1.5, respectively. For *L* = 400, the SNR for the G main effect is 0.050, 0.201 and 0.453 for ***γ***_*G*_ = 0.5, ***γ***_*G*_ = 1 and ***γ***_*G*_ = 1.5, respectively.

**FIGURE 10:**
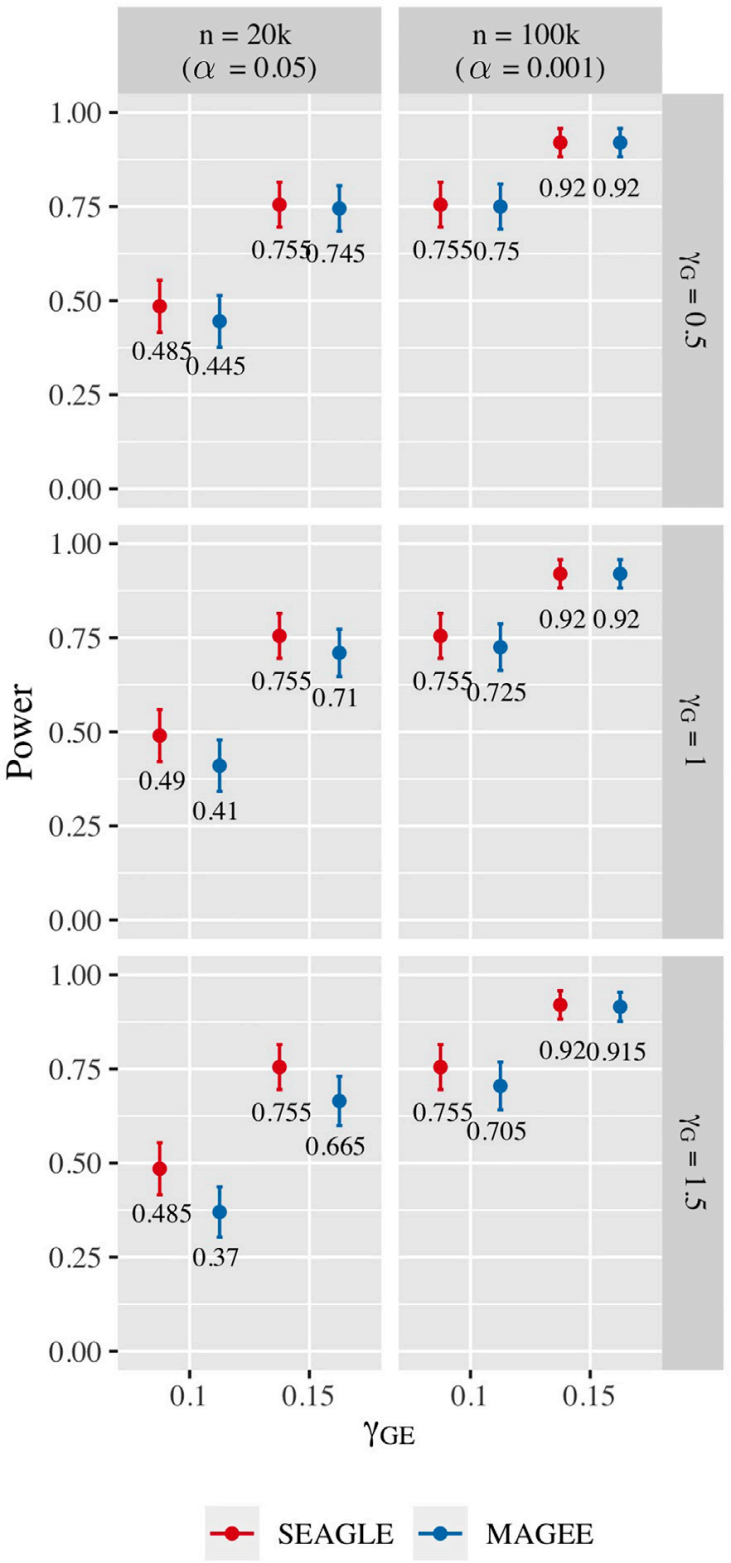
Power at *α* = 0.05 and *α* = 0.001 for p-values from fixed genetic effects model over *N* = 200 replicates with *n* = 20,000 and *n* = 100,000 observations, respectively, and *L* = 100 loci.

**FIGURE 11:**
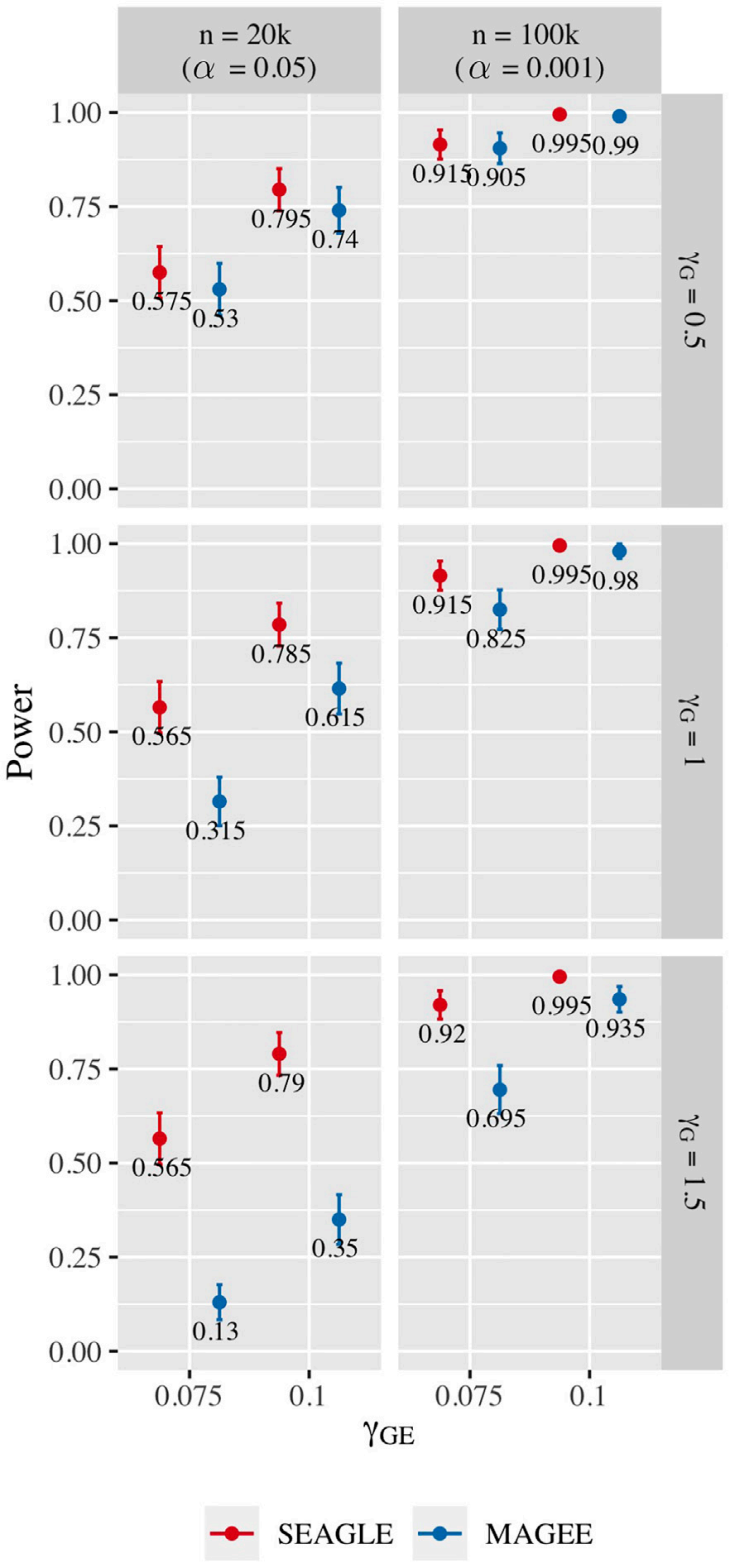
Power at *α* = 0.05 and *α* = 0.001 for p-values from fixed genetic effects model over *N* = 200 replicates with *n* = 20,000 and *n* = 100,000 observations, respectively, and *L* = 400 loci.

Figure 10 shows the power for *L* = 100 loci. At *n* = 20,000, SEAGLE exhibits better power than MAGEE at all combinations of *γ*_*G*_ and *γ*_*GE*_. Moreover, the difference in power increases for larger values of *γ*_*G*_ since MAGEE relies on the assumption that the G main effect size is small. At *n* = 100,000 and the same values of *γ*_*GE*_, we report the power at *α* = 0.001 instead of 0.05 because the power at *α* = 0.05 is near 1 for both methods. We see that both methods produce similar results although SEAGLE still outperforms MAGEE at slightly smaller values of *γ*_*GE*_. Figure 11 shows the power for *L* = 400 loci. Similar patterns of relative power performance are observed as in the case of *L* = 100, except that the power difference between SEAGLE and MAGEE is more pronounced in *L* = 400.

### 3.2 Application to the Taiwan Biobank Data

To illustrate the scalability of the G×E VC test using SEAGLE, we apply SEAGLE and MAGEE to the Taiwan Biobank (TWB) data. TWB is a nationwide biobank project initiated in 2012 and has recruited more than 15,995 individuals. Peripheral blood specimens were extracted and genotyped using the Affymetrix Genomewide Axiom TWB array, which was designed specifically for a Taiwanese population. We conduct the gene-based G×E analysis and evaluate the interaction between gene and physical activity (PA) status on body mass index (BMI), adjusting for age, sex and the top 10 principal components for population substructure. The PA status is a binary indicator for with/without regular physical activity. Our G×E analyses focuses on a subset of 11,664 unrelated individuals who have non-missing phenotype and covariate information. After PLINK quality control (i.e., removing SNPs with call rates < 0.95 or Hardy-Weinberg Equilibrium *p*-value < 10^−6^), there are 589,867 SNPs remaining, which are mapped to genes according to the gene range list “glist-hg19” from the PLINK Resources page at https://www.cog-genomics.org/plink/1.9/resources. There are a total of 13,260 genes containing > 1 SNPs for G×PA interaction analysis.

**FIGURE 12:**
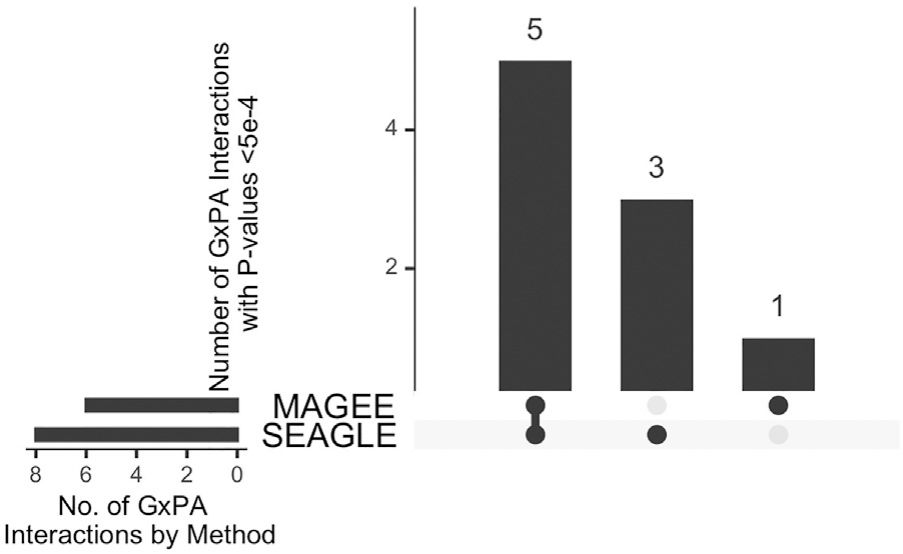
Upset plot of significant G×PA interactions identified by SEAGLE and MAGEE in the Taiwan Biobank at the 5 × 10^−4^ nominal level.

The median run time of SEAGLE and MAGEE is 2.4 and 1.3 s, respectively, Both SEAGLE and MAGEE do not find any significant G×PA interactions at the genome-wide Bonferroni threshold 0.05/13,260 = 3.77 × 10^−6^. We hence discuss the results using a less stringent threshold, i.e., 5 × 10^−4^ and summarize the results in Figure 12 and Supplementary Table S4. SEAGLE and MAGEE identify 8 and 6 G×PA interactions, respectively, among which 5 G×PA results are identified by both methods (Figure 12). The observation that SEAGLE identifies slightly more G×PA effects than MAGEE generally agrees with the simulation findings. We use the GeneCards Human Gene Database (www.genecards.org) (Stelzer et al., 2016) to explore the relevance of the identified genes with BMI or PA (see Supplementary Table S4). Two of the 5 commonly identified genes, i.e., *FCN2* and *OCM*, have non-zero relevance scores (i.e., 0.56 and 0.91, respectively). For the 3 genes identified by SEAGLE only, i.e., *ALOX5AP*, *BCLAF1* and *PCDH17*, their relevance scores are 6.16, 0.26 and 1.54, respectively. The expression of *ALOX5AP* has also been found associated with obesity and insulin resistance (Kaaman et al., 2006) as well as exercise-induced stress (Hilberg et al., 2005). On the other hand, *TBPL1* (identified by MAGEE only) is not in the GeneCards relevance list with BMI or PA.

## 4 Discussion

We introduced SEAGLE, a scalable exact algorithm for performing set-based G×E VC tests on large-scale biobank data. We achieve scalability and accuracy by applying modern numerical analysis techniques, which include avoiding the explicit formation of products and inverses of large matrices. Our numerical experiments illustrate that SEAGLE produces Type 1 error rates and power that are identical to those of the original VC test (Tzeng et al., 2011), while requiring a fraction of the computational cost. Moreover, SEAGLE is well-equipped to handle the very large dimensions required for analysis of large-scale biobank data.

State-of-the-art computational approaches such as MAGEE bypass the traditional time-consuming REML EM algorithm, and instead compute an approximation to the score-like test statistic by assuming that the G main effect size is small. In practice, however, the G main effect size is often unknown. Our numerical experiments illustrate that SEAGLE generally achieves better Type 1 error and power with comparable computation time.

In general, computational time differences between SEAGLE and MAGEE depend primarily on the effect size of the genetic main effect (G effect) and the size of the data, particularly the number of loci L. This is due to the fact that SEAGLE computes the score-like test statistic exactly and therefore requires an EM algorithm to estimate the main effect parameter *τ* and the noise effect parameter *σ*. The EM algorithm requires more time to converge when *τ* is small, particularly when *τ* is very close to zero. Furthermore, the EM algorithm also requires more computational time for data with larger dimensions, particularly as L grows larger. Despite these differences, the computational time for SEAGLE is at least comparable to that of MAGEE in all the many scenarios considered in this manuscript.

We highlight the fact that most of our timing experiments were performed on a 2013 Intel Core i5 laptop with a 2.70 GHz CPU and 16 GB RAM. Therefore, SEAGLE performs efficient and exact set-based G×E tests on biobank-scale data with *n* = 20,000 and *n* = 100,000 observations on ordinary laptops, without any need for high performance computational platforms. This makes SEAGLE broadly accessible to all researchers. Software for SEAGLE is publicly available as the SEAGLE package on GitHub (https://github.com/jocelynchi/SEAGLE), and is also available on the Comprehensive R Archive Network.

Recent studies suggested incorporating functional annotations can further enhance power and accuracy in set-based association analysis [e.g., STAAR (Li et al., 2020) and GAMBIT (Quick et al., 2020)], as a variant’s functional importance could better reflect the causal/null status of the variants than its MAF. The variant-specific annotation weights derived in these methods can also be incorporated into set-based GxE test (and hence SEAGLE). To do so, consider an *L* × 1 annotation weight vector **w**_*j*_ based on annotation class *j*, *j* = 1, …, *J* with *J* the total number of annotation classes, such as the scores derived based on tissue-specific regulatory annotations in Quick et al. (2020) or the estimated probability of being causal derived from the epigenetic annotation PCs in Li et al. (2020). We can first replace **G** by diag(**w**_*j*_)**G** in SEAGLE and obtain the association *p*-value of the target variant set for annotation class *j* (denoted by *p*_*j*_), *j* = 1, …, *J*. Then we combine these *p*_*j*_’s across annotation classes using the Cauchy Combination Test (Liu and Xie, 2020) or the Harmonic Mean *p*-value (Wilson, 2019), and obtain the final *p*-value of the variant set.

We conclude with a discussion of possible avenues for future extensions. First, the current SEAGLE framework only allows for quantitative traits. Extension to binary traits can be based on developing a scalable algorithm for the G×E VC test described in Zhao et al. (2015) using similar numerical analysis techniques, although the extension can be intricate due to the complexity of the EM algorithm for estimating the nuisance VCs involved with binary traits.

Second is the extension from a single environmental factor to a set of factors represented by $E\in R^{n\times q}$ with *q* > 1. The corresponding extension of Model (1) is

$$\begin{array}{ccc} y & = & X\beta_{X}+h_{E}+h_{G}+h_{GE}+\varepsilon ,\text{with }\\ & & h_{E}∼N\left(0,\tau_{E}\Sigma_{E}\right),\;h_{G}∼N\left(0,\tau_{G}\Sigma_{G}\right),\;h_{GE}∼N\left(0,\nu \Sigma_{GE}\right),\;\text{and }\varepsilon ∼N\left(0,\sigma I_{n}\right). \end{array}$$ (9)

Here $\Sigma_{E},\Sigma_{G},\Sigma_{GE}\in R^{n\times n}$ are variance matrices, where **Σ**_*E*_ = **EE** ^*T*^, **Σ**_*G*_ = **GG** ^*T*^ and **Σ**_*GE*_ is the element-wise product of **Σ**_*G*_ and **Σ**_*E*_. Adaptation of SEAGLE’s scalable REML EM algorithm to the EM algorithm in Wang et al. (2015b) takes care of estimating the nuisance VC parameters. Numerical analysis techniques analogous to the ones presented here will be the foundation for the efficient extension to multi-E factors.

Third is the extension from unrelated samples to family samples. With family samples, Model (1) will include an additional nuisance VC associated with kinship matrix to account for the within-family correlation. Similar scalable algorithms as for the multi-E analysis would also be useful for analyzing family samples.

The last is the extension to other types of kernels (Broadaway et al., 2015; Wang et al., 2015b) of the current random effects framework, which can be viewed as a special case of kernel machine regression with linear kernels. As in Lumley et al. (2018) and Wu and Sankararaman (2018), we will explore the potential of randomized numerical linear algebra, by drawing on the authors’ long standing expertise in the development of numerically stable, accurate and efficient randomized matrix algorithms (Eriksson-Bique et al., 2011; Ipsen and Wentworth, 2014; Wentworth and Ipsen, 2014; Holodnak and Ipsen, 2015; Saibaba et al., 2017; Holodnak et al., 2018; Drineas and Ipsen, 2019; Chi and Ipsen, 2021).

## Data Availability

The datasets for this article are not publicly available but requests to access the data from the Taiwan Biobank can be emailed to biobank@gate.sinica.edu.tw.

## References

[B1] Broadaway K. A. Duncan R. Conneely K. N. Almli L. M. Bradley B. Ressler K. J. (2015). Kernel Approach for Modeling Interaction Effects in Genetic Association Studies of Complex Quantitative Traits. Genet. Epidemiol. 39, 366–375. 10.1002/gepi.21901 25885490 PMC4469530

[B2] Chi J. T. Ipsen I. C. F. (2021). A Projector-Based Approach to Quantifying Total and Excess Uncertainties for Sketched Linear Regression. Inf. Inference, 1–23. 10.1093/imaiai/iaab016

[B3] Davies R. B. (1980). Algorithm AS 155: The Distribution of a Linear Combination of χ 2 Random Variables. Appl. Stat. 29, 323–333. 10.2307/2346911

[B4] Drineas P. Ipsen I. C. F. (2019). Low-Rank Matrix Approximations Do Not Need a Singular Value Gap. SIAM J. Matrix Anal. Appl. 40, 299–319. 10.1137/18m1163658

[B5] Eriksson-Bique S. Solbrig M. Stefanelli M. Warkentin S. Abbey R. Ipsen I. C. F. (2011). Importance Sampling for a Monte Carlo Matrix Multiplication Algorithm, with Application to Information Retrieval. SIAM J. Sci. Comput. 33, 1689–1706. 10.1137/10080659x

[B6] Favé M. J. Lamaze F. C. Soave D. Hodgkinson A. Gauvin H. Bruat V. (2018). Gene-by-environment Interactions in Urban Populations Modulate Risk Phenotypes. Nat. Commun. 9, 1–12. 10.1038/s41467-018-03202-2 29511166 PMC5840419

[B7] Golub G. H. Van Loan C. F. (2013). Matrix Computations 4th Edition, Vol. 4. Baltimore, Maryland, United States: The Johns Hopkins University Press.

[B8] Higham N. J. (2002). Accuracy and Stability of Numerical Algorithms. second edn. Philadelphia, PA: Society for Industrial and Applied Mathematics (SIAM).

[B9] Hilberg T. Deigner H. P. Möller E. Claus R. A. Ruryk A. Gläser D. (2005). Transcription in Response to Physical Stress-Clues to the Molecular Mechanisms of Exercise‐induced Asthma. FASEB j. 19, 1492–1494. 10.1096/fj.04-3063fje 16027142

[B10] Holodnak J. T. Ipsen I. C. F. (2015). Randomized Approximation of the Gram Matrix: Exact Computation and Probabilistic Bounds. SIAM J. Matrix Anal. Appl. 36, 110–137. 10.1137/130940116

[B11] Holodnak J. T. Ipsen I. C. F. Smith R. C. (2018). A Probabilistic Subspace Bound with Application to Active Subspaces. SIAM J. Matrix Anal. Appl. 39, 1208–1220. 10.1137/17m1141503

[B12] Hunter D. J. (2005). Gene-environment Interactions in Human Diseases. Nat. Rev. Genet. 6, 287–298. 10.1038/nrg1578 15803198

[B13] Ipsen I. C. F. Wentworth T. (2014). The Effect of Coherence on Sampling from Matrices with Orthonormal Columns, and Preconditioned Least Squares Problems. SIAM J. Matrix Anal. Appl. 35, 1490–1520. 10.1137/120870748

[B14] Kaaman M. Rydén M. Axelsson T. Nordström E. Sicard A. Bouloumié A. (2006). Alox5ap Expression, but Not Gene Haplotypes, Is Associated with Obesity and Insulin Resistance. Int. J. Obes. 30, 447–452. 10.1038/sj.ijo.0803147 16261187

[B15] Li X. Li Z. Zhou H. Gaynor S. M. Liu Y. Chen H. (2020). Dynamic Incorporation of Multiple In Silico Functional Annotations Empowers Rare Variant Association Analysis of Large Whole-Genome Sequencing Studies at Scale. Nat. Genet. 52, 969–983. 10.1038/s41588-020-0676-4 32839606 PMC7483769

[B16] Lin X. Lee S. Christiani D. C. Lin X. (2013). Test for Interactions between a Genetic Marker Set and Environment in Generalized Linear Models. Biostatistics 14, 667–681. 10.1093/biostatistics/kxt006 23462021 PMC3769996

[B17] Lin X. Lee S. Wu M. C. Wang C. Chen H. Li Z. (2016). Test for Rare Variants by Environment Interactions in Sequencing Association Studies. Biometrics 72, 156–164. 10.1111/biom.12368 26229047 PMC4733434

[B18] Liu H. Tang Y. Zhang H. H. (2009). A New Chi-Square Approximation to the Distribution of Non-negative Definite Quadratic Forms in Non-central normal Variables. Comput. Stat. Data Anal. 53, 853–856. 10.1016/j.csda.2008.11.025

[B19] Liu Y. Xie J. (2020). Cauchy Combination Test: a Powerful Test with Analytic P-Value Calculation under Arbitrary Dependency Structures. J. Am. Stat. Assoc. 115, 393–402. 10.1080/01621459.2018.1554485 33012899 PMC7531765

[B20] Lumley T. Brody J. Peloso G. Morrison A. Rice K. (2018). Fastskat: Sequence Kernel Association Tests for Very Large Sets of Markers. Genet. Epidemiol. 42, 516–527. 10.1002/gepi.22136 29932245 PMC6129408

[B21] Marceau R. Lu W. Holloway S. Sale M. M. Worrall B. B. Williams S. R. (2015). A Fast Multiple-Kernel Method with Applications to Detect Gene-Environment Interaction. Genet. Epidemiol. 39, 456–468. 10.1002/gepi.21909 26139508 PMC4544636

[B22] McAllister K. Mechanic L. E. Amos C. Aschard H. Blair I. A. Chatterjee N. (2017). Current Challenges and New Opportunities for Gene-Environment Interaction Studies of Complex Diseases. Am. J. Epidemiol. 186, 753–761. 10.1093/aje/kwx227 28978193 PMC5860428

[B23] Ottman R. (1996). Gene-Environment Interaction: Definitions and Study Design. Prev. Med. 25, 764–770. 10.1006/pmed.1996.0117 8936580 PMC2823480

[B24] Quick C. Wen X. Abecasis G. Boehnke M. Kang H. M. (2020). Integrating Comprehensive Functional Annotations to Boost Power and Accuracy in Gene-Based Association Analysis. Plos Genet. 16, e1009060. 10.1371/journal.pgen.1009060 33320851 PMC7737906

[B25] Ritz B. R. Chatterjee N. Garcia-Closas M. Gauderman W. J. Pierce B. L. Kraft P. (2017). Lessons Learned from Past Gene-Environment Interaction Successes. Am. J. Epidemiol. 186, 778–786. 10.1093/aje/kwx230 28978190 PMC5860326

[B26] Saibaba A. K. Alexanderian A. Ipsen I. C. F. (2017). Randomized Matrix-free Trace and Log-Determinant Estimators. Numer. Math. 137, 353–395. 10.1007/s00211-017-0880-z

[B27] Schaffner S. F. Foo C. Gabriel S. Reich D. Daly M. J. Altshuler D. (2005). Calibrating a Coalescent Simulation of Human Genome Sequence Variation. Genome Res. 15, 1576–1583. 10.1101/gr.3709305 16251467 PMC1310645

[B28] Stelzer G. Rosen N. Plaschkes I. Zimmerman S. Twik M. Fishilevich S. (2016). The Genecards Suite: from Gene Data Mining to Disease Genome Sequence Analyses. Curr. Protoc. Bioinformatics 54, 1–33. 10.1002/cpbi.5 27322403

[B29] Su Y.-R. Di C.-Z. Hsu L. (2017). A Unified Powerful Set-Based Test for Sequencing Data Analysis of Gxe Interactions. Biostat 18, 119–131. 10.1093/biostatistics/kxw034 PMC5255050 27474101

[B30] Sulc J. Winkler T. W. Heid I. M. Kutalik Z. Wood A. R. Frayling T. M. (2020). Heterogeneity in Obesity: Genetic Basis and Metabolic Consequences. Curr. Diab Rep. 20, 1–13. 10.1007/s11892-020-1285-4 31970540

[B31] Tzeng J.-Y. Zhang D. Pongpanich M. Smith C. McCarthy M. I. Sale M. M. (2011). Studying Gene and Gene-Environment Effects of Uncommon and Common Variants on Continuous Traits: a Marker-Set Approach Using Gene-Trait Similarity Regression. Am. J. Hum. Genet. 89, 277–288. 10.1016/j.ajhg.2011.07.007 21835306 PMC3155192

[B32] Wang X. Lim E. Liu C. T. Sung Y. J. Rao D. C. Morrison A. C. (2020). Efficient Gene-Environment Interaction Tests for Large Biobank‐scale Sequencing Studies. Genet. Epidemiol. 44, 908–923. 10.1002/gepi.22351 32864785 PMC7754763

[B33] Wang Z. Maity A. Luo Y. Neely M. L. Tzeng J.-Y. (2015a). Complete Effect-Profile Assessment in Association Studies with Multiple Genetic and Multiple Environmental Factors. Genet. Epidemiol. 39, 122–133. 10.1002/gepi.21877 25538034 PMC4314365

[B34] Wang Z. Maity A. Luo Y. Neely M. L. Tzeng J.-Y. (2015b). Complete Effect-Profile Assessment in Association Studies with Multiple Genetic and Multiple Environmental Factors. Genet. Epidemiol. 39, 122–133. 10.1002/gepi.21877 25538034 PMC4314365

[B35] Wentworth T. Ipsen I. C. F. (2014). Kappa_SQ: A Matlab Package for Randomized Sampling of Matrices with Orthonormal Columns. arXiv.

[B36] Wilson D. J. (2019). The Harmonic Mean P-Value for Combining Dependent Tests. Proc. Natl. Acad. Sci. USA 116, 1195–1200. 10.1073/pnas.1814092116 30610179 PMC6347718

[B37] Wu Y. Sankararaman S. (2018). A Scalable Estimator of Snp Heritability for Biobank-Scale Data. Bioinformatics 34, i187–i194. 10.1093/bioinformatics/bty253 29950019 PMC6022682

[B38] Zhao G. Marceau R. Zhang D. Tzeng J.-Y. (2015). Assessing Gene-Environment Interactions for Common and Rare Variants with Binary Traits Using Gene-Trait Similarity Regression. Genetics 199, 695–710. 10.1534/genetics.114.171686 25585620 PMC4349065

