# Supplementary material for: SEAGLE: A Scalable Exact Algorithm for Large-Scale Set-Based Gene-Environment Interaction Tests in Biobank Data
Source: Front Genet. 2021 Nov 2;12:710055. doi: 10.3389/fgene.2021.710055 (PMC8593472; doi:10.3389/fgene.2021.710055)
Supplement: Supplementary file 1 [file DataSheet1.pdf]

Supplementary Material

1 APPENDIX: RESTRICTED MAXIMUM LIKELIHOOD (REML) EM ALGORITHM FOR ESTIMATING VARIANCE COMPONENTS

Under the null hypothesis $H_0 : \nu = 0$ and the assumptions that $\tau > 0$ and $\sigma > 0$, model (1) becomes

$$\mathbf{y} \quad = \quad \widetilde{\mathbf{X}}\boldsymbol{\beta} + \mathbf{G}\mathbf{b} + \boldsymbol{\epsilon}, \tag{S1}$$

where $\mathbf{b} \sim \mathrm{N}(\mathbf{0}, \tau\,\mathbf{I}_L)$ and $\boldsymbol{\epsilon} \sim \mathrm{N}(\mathbf{0}, \sigma\,\mathbf{I}_n)$ are uncorrelated. Assume that $\widetilde{\mathbf{X}}$ has full column rank so that $\mathrm{rank}(\widetilde{\mathbf{X}}) = P$. Let $\mathbf{u} = \mathbf{A}^T\mathbf{y}$ such that $\mathbf{A}\mathbf{A}^T = \mathbf{I} - \widetilde{\mathbf{X}}(\widetilde{\mathbf{X}}^T\widetilde{\mathbf{X}})^{-1}\widetilde{\mathbf{X}}^T$ and $\mathbf{A}^T\mathbf{A} = \mathbf{I}_{n-P}$. Then

$$\begin{aligned} \mathbf{u} &= \mathbf{A}^T\mathbf{y} \\ &= \mathbf{A}^T\mathbf{G}\mathbf{b} + \mathbf{A}^T\boldsymbol{\epsilon} \end{aligned}$$

so that $\mathbf{u}\,|\,\mathbf{b}$ does not depend on the unobserved fixed effects parameter $\boldsymbol{\beta}$.

Following the work in (Tzeng et al., 2011), we consider an expectation-maximization (EM) algorithm based on the restricted maximum likelihood for the observed data $\mathbf{u}$ and missing data $\mathbf{b}$ with the following complete data log likelihood

$$\log f(\mathbf{u}, \mathbf{b}; \tau, \sigma) \quad = \quad \log f(\mathbf{u}\,|\,\mathbf{b}; \tau, \sigma) + \log f(\mathbf{b}; \tau, \sigma). \tag{S2}$$

For the first term on the right-hand side of (S2), we use the fact that the conditional distribution of $\mathbf{u}$ given $\mathbf{b}$ is

$$\mathbf{u}\,|\,\mathbf{b} \sim \mathrm{N}(\mathbf{A}^T\mathbf{G}\mathbf{b}, \sigma\,\mathbf{I}_{n-P})$$

so that

$$f(\mathbf{u}\,|\,\mathbf{b}; \tau, \sigma) \quad = \quad (2\pi)^{-\frac{n-P}{2}}\,|\sigma\,\mathbf{I}_{n-P}|^{-\frac{1}{2}}\,\exp\left\{-\frac{1}{2\sigma}\|\mathbf{u} - \mathbf{A}^T\mathbf{G}\mathbf{b}\|_2^2\right\}$$

and

$$\log f(\mathbf{u}\,|\,\mathbf{b}; \tau, \sigma) \quad \propto \quad -\frac{n-P}{2}\log\sigma - \frac{1}{2\sigma}\|\mathbf{u} - \mathbf{A}^T\mathbf{G}\mathbf{b}\|_2^2. \tag{S3}$$

For the second term on the right-hand side of (S2), we have

$$f(\mathbf{b}; \tau, \sigma) \quad = \quad (2\pi)^{-\frac{L}{2}}\,|\tau\,\mathbf{I}_L|^{-\frac{1}{2}}\,\exp\left\{-\frac{1}{2\tau}\|\mathbf{b}\|_2^2\right\}$$

so that

$$\log f(\mathbf{b}; \tau, \sigma) \quad \propto \quad -\frac{L}{2}\log\tau - \frac{1}{2\tau}\|\mathbf{b}\|_2^2. \tag{S4}$$

Inserting (S3) and (S4) into (S2) gives

$$\log f(\mathbf{u}, \mathbf{b}; \tau, \sigma) \quad \propto \quad -\frac{n-P}{2} \log \sigma - \frac{1}{2\sigma} \|\mathbf{u} - \mathbf{A}^T \mathbf{G} \mathbf{b}\|_2^2 - \frac{L}{2} \log \tau - \frac{1}{2\tau} \|\mathbf{b}\|_2^2.$$

Therefore, $Q(\tau, \sigma; \hat{\tau}_t, \hat{\sigma}_t)$ in the expectation step (E-step) is

$$\begin{aligned} Q(\tau, \sigma; \hat{\tau}_t, \hat{\sigma}_t) \quad &\equiv \quad \mathbb{E}\left[\log f(\mathbf{u}, \mathbf{b}; \tau, \sigma) \,|\, \mathbf{u}; \hat{\tau}_t, \hat{\sigma}_t\right] \\ &\propto \quad -\frac{n-P}{2} \log \sigma - \frac{1}{2\sigma}\, \mathbb{E}\left[\|\mathbf{u} - \mathbf{A}^T \mathbf{G} \mathbf{b}\|_2^2 \,|\, \mathbf{u}; \hat{\tau}_t, \hat{\sigma}_t\right] \\ &\quad\;\; -\frac{L}{2} \log \tau - \frac{1}{2\tau}\, \mathbb{E}\left[\|\mathbf{b}\|_2^2 \,|\, \mathbf{u}; \hat{\tau}_t, \hat{\sigma}_t\right]. \end{aligned}$$

To obtain the distribution of $\mathbf{b} \,|\, \mathbf{u}$, we recall the fact that for any $\mathbf{g}_1 \in \mathbb{R}^q$ and $\mathbf{g}_2 \in \mathbb{R}^{N-q}$ such that

$$\begin{bmatrix} \mathbf{g}_1 \\ \mathbf{g}_2 \end{bmatrix} \sim \mathrm{N}\left(\begin{bmatrix} \boldsymbol{\mu}_1 \\ \boldsymbol{\mu}_2 \end{bmatrix}, \begin{bmatrix} \Sigma_{11} & \Sigma_{12} \\ \Sigma_{21} & \Sigma_{22} \end{bmatrix} \right),$$

the distribution of $\mathbf{g}_1 \,|\, \mathbf{g}_2$ is

$$\mathbf{g}_1 \,|\, \mathbf{g}_2 \sim \mathrm{N}(\boldsymbol{\mu}_1 + \Sigma_{12}\Sigma_{22}^{-1}(\mathbf{g}_2 - \boldsymbol{\mu}_2), \Sigma_{11} - \Sigma_{12}\Sigma_{22}^{-1}\Sigma_{21}).$$

Since the covariance between $\mathbf{b}$ and $\mathbf{u}$ is

$$\begin{aligned} \mathbb{C}\mathrm{ov}(\mathbf{b}, \mathbf{u}) \quad &= \quad \mathbb{E}[\mathbf{b}(\mathbf{u} - \mathbb{E}[\mathbf{u}])^T] \\ &= \quad \mathbb{E}[\mathbf{b}\{\mathbf{A}^T(\widetilde{\mathbf{X}}\boldsymbol{\beta} + \mathbf{G}\mathbf{b} + \boldsymbol{\epsilon}) - \mathbf{0}\}^T] \\ &= \quad \mathbb{E}[\mathbf{b}\,\mathbf{b}^T \mathbf{G}^T \mathbf{A}] \\ &= \quad \mathbb{V}\mathrm{ar}(\mathbf{b})\mathbf{G}^T\mathbf{A} = \tau\, \mathbf{G}^T \mathbf{A} = \Sigma_{12}, \end{aligned}$$

the joint distribution of $\mathbf{b}$ and $\mathbf{u}$ is

$$\begin{bmatrix} \mathbf{b} \\ \mathbf{u} \end{bmatrix} \sim \mathrm{N}\left(\begin{bmatrix} \mathbf{0} \\ \mathbf{0} \end{bmatrix}, \begin{bmatrix} \tau\, \mathbf{I}_L & \tau\, \mathbf{G}^T \mathbf{A} \\ \tau\, \mathbf{A}^T \mathbf{G} & \tau\, \mathbf{A}^T \mathbf{K}_G \mathbf{A} + \sigma\, \mathbf{I}_{n-P} \end{bmatrix} \right)$$

and we have

$$\mathbf{b} \,|\, \mathbf{u} \sim \mathrm{N}(\tau\, \mathbf{G}^T \mathbf{A} \mathbf{R}^{-1} \mathbf{u}, \tau\, \mathbf{I}_L - \tau^2\, \mathbf{G}^T \mathbf{A} \mathbf{R}^{-1} \mathbf{A}^T \mathbf{G}),$$

where $\mathbf{R} = \tau\, \mathbf{A}^T \mathbf{K}_G \mathbf{A} + \sigma\, \mathbf{I}_{n-P}$. Therefore, in the maximization step (M-step), we have

$$\frac{\partial Q}{\partial \sigma} \quad = \quad -\frac{n-P}{2\sigma} + \frac{1}{2\sigma^2}\, \mathbb{E}\left[\|\mathbf{u} - \mathbf{A}^T \mathbf{G} \mathbf{b}\|_2^2 \,|\, \mathbf{u}; \hat{\tau}_t, \hat{\sigma}_t\right]$$

so that

$$\begin{aligned} \hat{\sigma}_{t+1} \quad &= \quad \frac{1}{n-P}\, \mathbb{E}\left[\|\mathbf{u} - \mathbf{A}^T \mathbf{G} \mathbf{b}\|_2^2 \,|\, \mathbf{u}; \hat{\tau}_t, \hat{\sigma}_t\right] \\ &= \quad \frac{1}{n-P}\left[\|\mathbf{u} - \hat{\tau}_t\, \mathbf{A}^T \mathbf{K}_G \mathbf{A} \mathbf{R}^{-1} \mathbf{u}\|_2^2 + \hat{\tau}_t\ \mathrm{trace}(\mathbf{A}^T \mathbf{K}_G \mathbf{A} - \hat{\tau}_t\, \mathbf{A}^T \mathbf{K}_G \mathbf{A} \mathbf{R}^{-1} \mathbf{A}^T \mathbf{K}_G \mathbf{A})\right] \end{aligned}$$

since $\mathbb{E}[\mathbf{u} - \mathbf{A}^T\mathbf{G}\mathbf{b} \,|\, \mathbf{u}] = \mathbf{u} - \tau\mathbf{A}^T\mathbf{K}_G\mathbf{A}\mathbf{R}^{-1}\mathbf{u}$ and $\mathbb{V}\mathrm{ar}(\mathbf{u} - \mathbf{A}^T\mathbf{G}\mathbf{b} \,|\, \mathbf{u}) = \tau\,\mathbf{A}^T\mathbf{K}_G^T\mathbf{A} - \tau^2\mathbf{A}^T\mathbf{K}_G\mathbf{A}\mathbf{R}^{-1}\mathbf{A}^T\mathbf{K}_G\mathbf{A}$. For the first term on the right, we have

$$\begin{aligned}
\mathbf{u} - \hat{\tau}_t\,\mathbf{A}^T\mathbf{K}_G\mathbf{A}\mathbf{R}^{-1}\mathbf{u} &= (\mathbf{R}\mathbf{R}^{-1} - \hat{\tau}_t\,\mathbf{A}^T\mathbf{K}_G\mathbf{A}\mathbf{R}^{-1})\mathbf{u} \\
&= (\mathbf{R} - \hat{\tau}_t\,\mathbf{A}^T\mathbf{K}_G\mathbf{A})\mathbf{R}^{-1}\mathbf{u} \\
&= \hat{\sigma}_t\,\mathbf{R}^{-1}\mathbf{u}.
\end{aligned}$$

For the second term on the right, we have

$$\begin{aligned}
\hat{\tau}_t\ \mathrm{trace}(\mathbf{A}^T\mathbf{K}_G\mathbf{A} - \hat{\tau}_t\,\mathbf{A}^T\mathbf{K}_G\mathbf{A}\mathbf{R}^{-1}\mathbf{A}^T\mathbf{K}_G\mathbf{A}) &= \mathrm{trace}\left([\mathbf{R} - \hat{\tau}_t\,\mathbf{A}^T\mathbf{K}_G\mathbf{A}]\hat{\tau}_t\,\mathbf{R}^{-1}\mathbf{A}^T\mathbf{K}_G\mathbf{A}\right) \\
&= \hat{\sigma}_t\,\hat{\tau}_t\ \mathrm{trace}(\mathbf{R}^{-1}\mathbf{A}^T\mathbf{K}_G\mathbf{A}) \\
&= \hat{\sigma}_t\,\hat{\tau}_t\ \mathrm{trace}(\mathbf{G}^T\mathbf{A}\mathbf{R}^{-1}\mathbf{A}^T\mathbf{G}).
\end{aligned}$$

Inserting these simplifications into the update for $\hat{\sigma}_t$ gives us

$$\hat{\sigma}_{t+1} = \frac{1}{n-P}\left[\|\hat{\sigma}_t\,\widehat{\mathbf{R}}^{-1}\mathbf{u}\|_2^2 + \hat{\sigma}_t\,\hat{\tau}_t\ \mathrm{trace}(\mathbf{G}^T\mathbf{A}\widehat{\mathbf{R}}^{-1}\mathbf{A}^T\mathbf{G})\right], \tag{S5}$$

where $\widehat{\mathbf{R}} = \hat{\tau}_t\,\mathbf{A}^T\mathbf{K}_G\mathbf{A} + \hat{\sigma}_t\,\mathbf{I}_{n-P}$.

Additionally, we have

$$\frac{\partial Q}{\partial \tau} = -\frac{L}{2\tau} + \frac{1}{2\tau^2}\,\mathbb{E}[\|\mathbf{b}\|_2^2 \,|\, \mathbf{u}; \hat{\tau}_t, \hat{\sigma}_t]$$

so that

$$\begin{aligned}
\hat{\tau}_{t+1} &= \frac{1}{L}\,\mathbb{E}[\|\mathbf{b}\|_2^2 \,|\, \mathbf{u}; \hat{\tau}_t, \hat{\sigma}_t] \\
&= \frac{1}{L}\left[\hat{\tau}_t^2\,\|\mathbf{G}^T\mathbf{A}\widehat{\mathbf{R}}^{-1}\mathbf{u}\|_2^2 + \mathrm{tr}(\hat{\tau}_t\,\mathbf{I}_L - \hat{\tau}_t^2\mathbf{G}^T\mathbf{A}\widehat{\mathbf{R}}^{-1}\mathbf{A}^T\mathbf{G})\right].
\end{aligned} \tag{S6}$$

2 SUPPLEMENTARY TABLES AND FIGURES

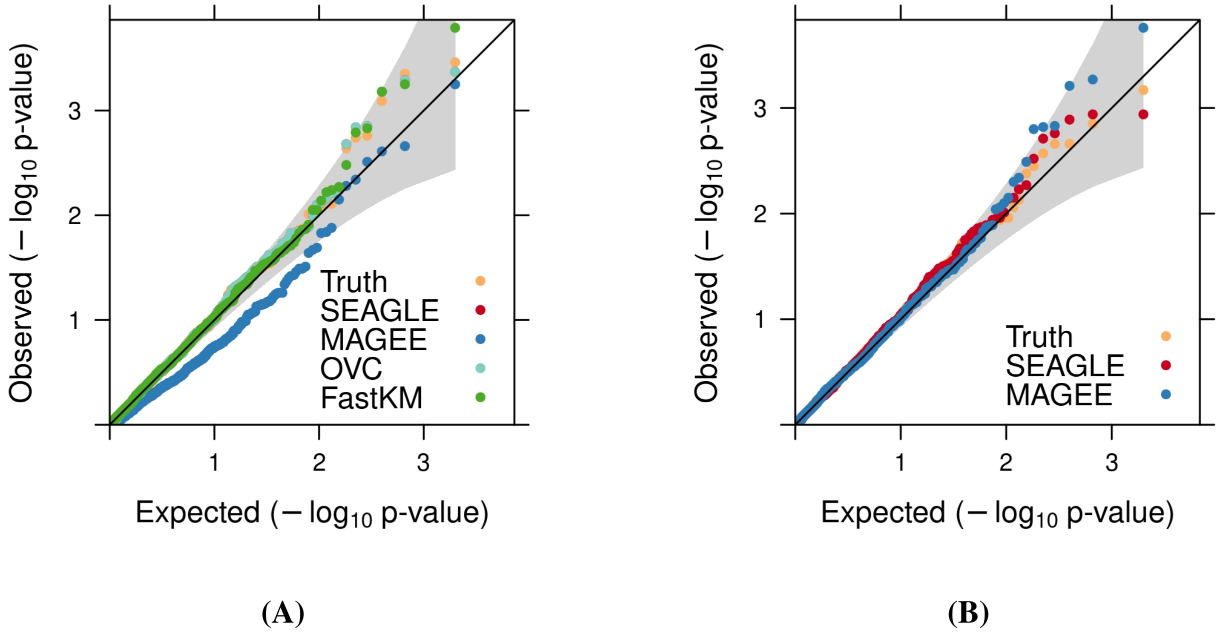

Figure S1. *Quantile-Quantile plots depicting p-values over* $N = 1{,}000$ *replicates for* $n = 5{,}000$ *observations and* $L = 100$ *loci under the null hypothesis in the random effects simulations. (A)* $\tau = 1$ *and (B)* $\tau = 0.01$.

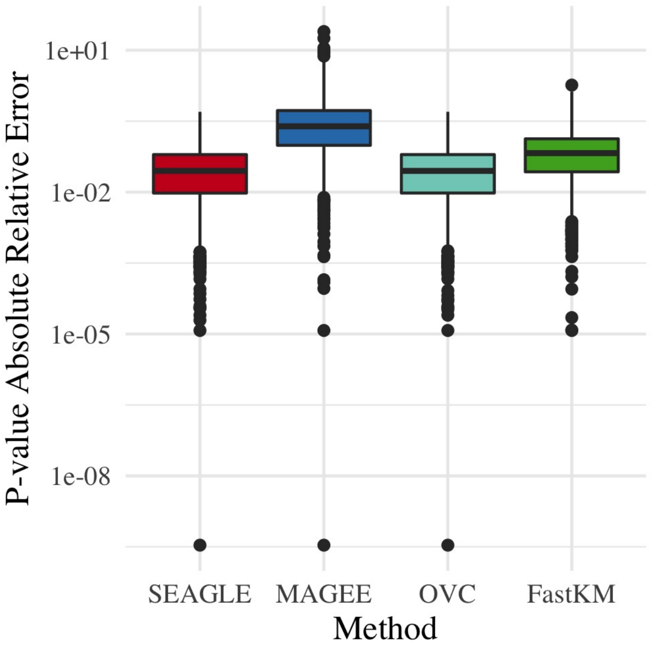

Figure S2. *Absolute relative error of the p-values obtained from different GxE VC tests, compared to the "Truth" p-values. Results are obtained with $\tau = \sigma = 1$ under $H_0 : \nu = 0$ over $N = 1{,}000$ replicates with $n = 5{,}000$ observations and $L = 100$ loci.*

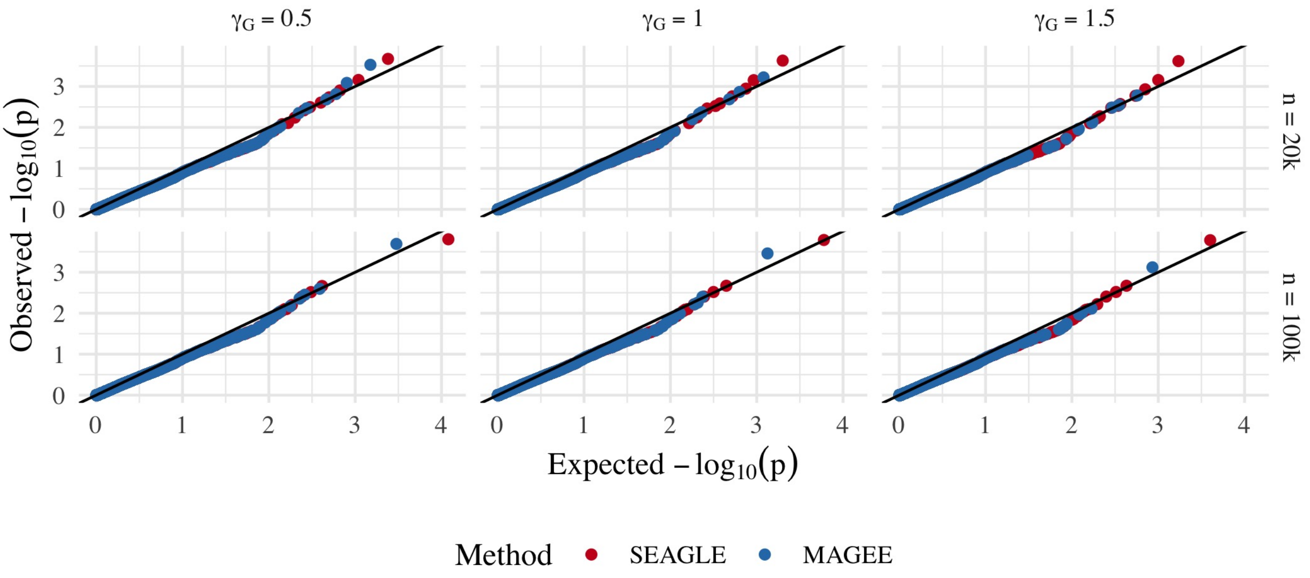

Figure S3. *Quantile-quantile plots for p-values obtained over $N = 1{,}000$ replicates for $n = 20{,}000$ and $n = 100{,}000$ observations and $L = 100$ loci in the fixed effects simulations.*

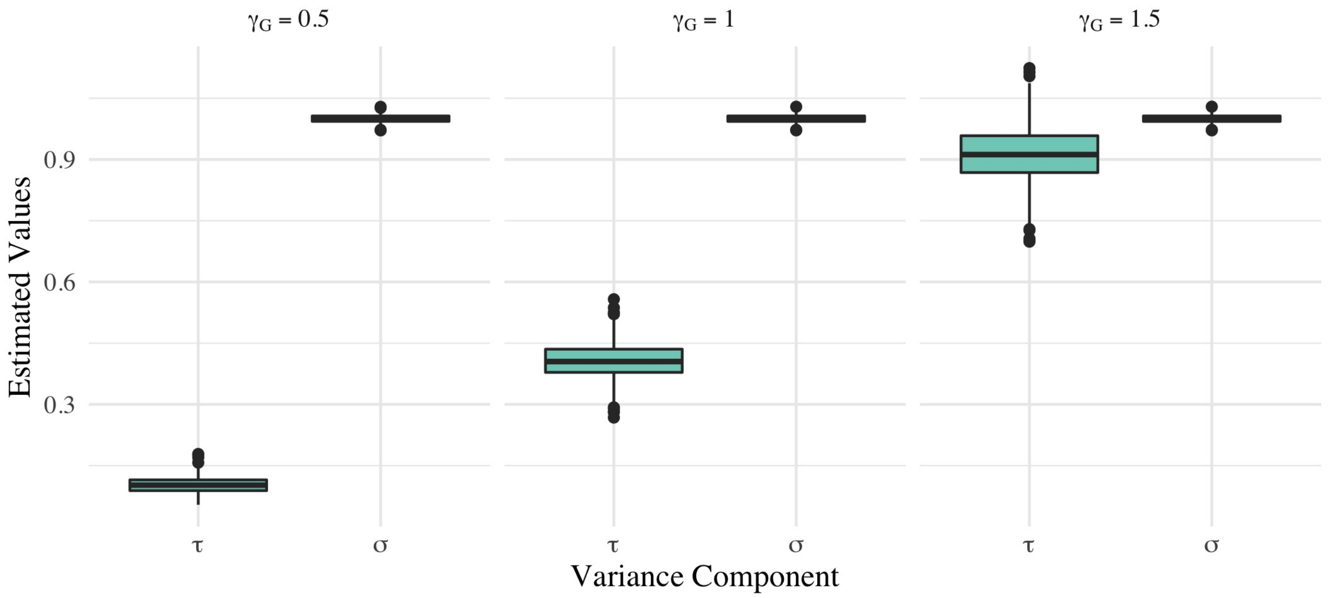

Figure S4. *Estimated values for τ and σ over $N = 1{,}000$ replicates with $n = 20{,}000$ observations and $L = 100$ loci.*

Table S1. *P-values of the G×PA effects (i.e., interactions between genes and physical activity (PA)) on body mass index (BMI), identified by SEAGLE and MAGEE in the Taiwan Biobank study at the 5×10^{-4} nominal level. Relevance scores are obtained from GeneCards human gene database (www.genecards.org) using multiword search with string "'body mass index' OR obesity OR 'physical activity'".*

Gene	SEAGLE	MAGEE	Relevance Score with BMI, obesity or PA
ALOX5AP	4.33E-04	-	6.16
BCLAF1	1.83E-04	-	0.26
CBLN2	3.05E-05	3.71E-05	-
FCN2	4.08E-04	2.70E-04	0.56
FOXR1	3.22E-04	1.53E-04	-
LOC338694	1.34E-04	1.83E-04	-
OCM	4.18E-04	8.58E-05	0.91
PCDH17	3.64E-04	-	1.54
TBPL1	-	2.24E-04	-

REFERENCES

Tzeng JY, Zhang D, Pongpanich M, Smith C, McCarthy MI, Sale MM, et al. Studying gene and gene-environment effects of uncommon and common variants on continuous traits: a marker-set approach using gene-trait similarity regression. *The American Journal of Human Genetics* **89** (2011) 277–288.
